# Analysis of the effect of CCR7 on the microenvironment of mouse oral squamous cell carcinoma by single-cell RNA sequencing technology

**DOI:** 10.1186/s13046-024-03013-y

**Published:** 2024-03-27

**Authors:** Zengxu Wang, Keith L. Kirkwood, Yao Wang, Weidong Du, Shanfeng Lin, Wanhang Zhou, Cong Yan, Jiaxing Gao, Zhenning Li, Changfu Sun, Fayu Liu

**Affiliations:** 1 Department of Oral Maxillofacial-Head and Neck Surgery, School and Hospital of Stomatology, China Medical University, Liaoning Provincial Key Laboratory of Oral Diseases, 117 Nanjing North Road, Heping District, Shenyang, Liaoning 110002 People’s Republic of China; 2 Department of Oral Biology, School of Dental Medicine, University at Buffalo, NY Buffalo, 14214-8006 USA

**Keywords:** OSCC, scRNA-seq, TME, CCR7

## Abstract

**Background:**

Studies have shown that CCR7, an important inflammatory factor, can promote the proliferation and metastasis of oral squamous cell carcinoma (OSCC), but its role in the tumor microenvironment (TME) remains unclear. This paper explores the role of CCR7 in the TME of OSCC.

**Methods:**

In this work, we constructed CCR7 gene knockout mice and OSCC mouse models. Single-cell RNA sequencing (scRNA-seq) and bioinformatics were used to analyze the differences in the OSCC microenvironment between three CCR7 gene knockout mice (KO) and three wild-type mice (WT). Immunohistochemistry, immunofluorescence staining, and flow cytometry were used to analyze the expression of key genes in significantly different cell types between the KO and WT groups. An in vitro experiment was used to verify the effect of CCR7 on M2 macrophage polarization.

**Results:**

In the mouse OSCC models, the tumor growth rate in the KO group was significantly lower than that in the WT group. Eight main cell types (including tumor cells, fibroblasts, macrophages, granulocytes, T cells, endothelial cells, monocytes, and B cells) were identified by Seurat analysis. The scRNA-seq results showed that the proportion of tumor cells was lower, but the proportion of inflammatory cells was significantly higher in the KO group than in the WT group. CellPhoneDB analysis results indicated a strong interaction relationship between tumor cells and macrophages, T cells, fibroblasts, and endothelial cells. Functional enrichment results indicated that the expression level of the *Dusp1* gene in the KO group was generally higher than that in the WT group in various cell types. Macrophage subclustering results indicated that the proportion of M2 macrophages in the KO group was lower than that in the WT group. In vitro experimental results showed that CCR7 can promote M2 macrophage polarization, thus promoting the proliferation, invasion and migration of OSCC cells.

**Conclusions:**

CCR7 gene knockout can significantly inhibit the growth of mouse oral squamous cell carcinoma by promoting the polarization of M2 macrophages.

**Supplementary Information:**

The online version contains supplementary material available at 10.1186/s13046-024-03013-y.

## Background

Oral cancer is the sixth most common malignancy worldwide [1]. More than 90% of oral cancers originate from oral squamous epithelial tissues, and this type of cancer is widely known as oral squamous cell carcinoma (OSCC) [2]. Currently, primary treatment options include surgical resection, chemotherapy and radiotherapy, which carry increased patient morbidity. Despite advances in these traditional therapeutic approaches, the five-year survival rate for patients with oral squamous cell carcinoma remains below 70% [3]. In recent years, immunotherapy has received great attention and achieved good results in a variety of malignant tumors. For instance, atezolizumab combined with nab-paclitaxel was approved for the treatment of patients with unresectable locally advanced or metastatic triple-negative breast cancer (TNBC) whose tumors express PD-L1 [4]. However, although anti-PD-1/PD-L1 antibodies have been approved by the Food and Drug Administration (FDA) for the treatment of OSCC, the overall response rate is still low [5, 6]. Therefore, finding specific immunotherapy targets for OSCC has become increasingly critical for patient managment.

The tumor microenvironment (TME) consists of nontumor cells, vascular and lymphatic endothelial cells, various immune cells [including lymphocytes, tumor-associated macrophages (TAMs), granulocytes, and tumor-associated fibroblasts (CAFs)] and surrounding related metabolites [7, 8]. In nearly 50% of OSCC cases, TAMs are the main immune cell population of the OSCC TME that can promote or inhibit the proliferation and invasion of OSCC cells according to their activation status (M1 or M2) [9, 10]. Generally, M1 macrophages play an antitumor role through the production of proinflammatory cytokines (IL-2, IL-23, TNF-α, etc.) [11, 12]. M2 macrophages produce anti-inflammatory cytokines such as IL-10 and TGF-β, thereby promoting tumor immune escape [13, 14]. In addition, in the TME, T cells significantly affect tumor occurrence and progression, where the overall survival and relapse-free survival rates are positively correlated with CD4 and CD8 T-cell levels in OSCC [15, 16]. Collectively, these data indicate that the occurrence, growth, and metastasis of OSCC are closely related to the immune cells in the TME [17]. However, many specific mechanisms of the interaction between OSCC and the TME remain unclear.

Chemokine receptor 7 (CCR7) is a potent G-protein-coupled receptor (GPCR) that performs its biological function by interacting with its two ligands, CCL19 and CCL22 [18]. Our previous studies have shown that CCR7 can promote the proliferation, invasion, and migration of OSCC cells through PI3K/AKT/mTOR, PLC/PKC, the MAPK family, pyk2 and several other molecules [19–22]. In addition, studies have shown that CCR7 deficiency can significantly delay PyMT-driven primary mammary tumorigenesis [23]. However, the effect of CCR7 on the microenvironment of OSCC is still unclear.

For this study, we obtained CCR7 gene knockout mice and analyzed OSCC microenvironment changes by single-cell transcriptomic analyses. Subsequently, in vitro experiments were conducted to further confirm the effect of CCR7 on macrophage polarization that directly impacts the TME and resistance to immunotherapies.

## Materials and methods

### Cell culture

C57BL/6-derived mouse oral cancer cells (MOC-1 and MOC-2) were purchased from Kerafast (USA) and cultured in HyClone Iscove’s modified Dulbecco’s medium (IMDM)/HyClone Ham’s Nutrient Mixture F12 at a 2:1 mixture with 5% FCS (Fisher, Scientific, Houston, TX), 1% penicillin/streptomycin, 1% amphotericin, 5 ng/ml epidermal growth factor (EGF, Millipore, Billerica, MA), 5 μg/ml insulin (Sigma, Chemical, ST. Louis, MO), and 400 ng/ml hydrocortisone as described previously [24]. PCI-37B and PCI-4B (human head and neck squamous cell carcinoma cell line) cells were donated by the University of Pittsburgh (USA) and cultured in DMEM with 10% FBS and 1% penicillin/streptomycin at 37 °C. THP-1 (human leukemia monocytic cell line) cells were purchased from Shanghai Cell Collection of Chinese Academy of Sciences and cultured in RPMI 1640 with 10% FBS, 1% penicillin/streptomycin and 0.05 mM β-mercaptoethanol at 37 °C.

### Macrophage induction

Induction of the human macrophage line THP-1 was performed as previously reported [25, 26]. Briefly, THP-1 cells were cultured with PMA (100 ng/ml) for 24 h and then in RPMI 1640 complete culture medium for 48 h to induce M0 macrophages. M0 macrophages were cultured with IL-4 and IL-13 (20 ng/ml) for 48 h to induce M2 macrophages.

### CCR7 knockout mouse construction and OSCC model construction

CCR7 knockout mice were purchased from Cyagen Biosciences (Suzhou, China). Because OSCC has the same growth trend between the flank and oral cavity of mice and the flank tumor is easier to measure, previous research generally used the mouse flank to construct an OSCC tumor model [27]. MOC-1 cells (1.0 × 10^5^) or MOC-2 cells (1.0 × 10^6^) in 100 µl of PBS were implanted subcutaneously into *CCR7*^−/−^ (KO) and wild-type (WT) mouse flanks. The length and width of the tumors were measured by using digital calipers every two days starting from the sixth day after the injection of MOC-1 or MOC-2 cells, and the tumor volume was calculated by (length × width^2^)/2. Then, the mice were sacrificed when the tumor volume reached 1500 mm^3^, and the tumor tissues were used for subsequent single-cell sequencing, immunofluorescence and flow cytometry analysis.

### Single-cell RNA sequencing analyses

According to the manufacturer’s instructions, single-cell RNA sequencing libraries were constructed using a Single Cell 3’ Library and Gel Bead Kit V3 (10 × Genomics, 1,000,075, Capital Bio Technology, Beijing, China). The cells were clustered by Seurat 3.0 (R package). Dimensionality reduction was performed using PCA, and visualization was realized by t-SNE. GO, KEGG and Reactome enrichment were performed using KOBAS software with Benjamini‒Hochberg multiple testing adjustment according to the top 50 marker genes of each cluster. GSEA (Gene Set Enrichment Analysis) was performed by using GSEA software (version 2.2.2.4), which uses predefined gene sets from the Molecular Signatures Database (MSigDB v6.2) [28]. Gene set variation analysis (GSVA) was performed by using the GSVA R package based on the top 50 differential marker genes between the KO and WT groups. Gene sets came from the Molecular Signatures Database (MSigDB v6.2). Single-cell trajectories were built with the Monocle 2 R package that introduced pseudotime. SingleR (https://bioconductor.org/packages/devel/bioc/html/SingleR.html) was used to match the cell type of each single cell referring to the annotation of mouse cell types from Benayoun [29] and finally obtain the most likely cell type for each cell. Cell interaction analysis was performed using CellPhoneDB [30] between the KO and WT groups. Cell interactions were considered relevant if the *p* value of ligand–receptor pairs was less than 0.05.

### TCGA database analysis

Gene Express Profiling Interactive Analysis (GEPIA2) was used to examine the expression analysis and survival analysis of oral squamous cell carcinoma for the significantly different genes between the WT and KO groups in this work [31].

### Immunohistochemistry

Mouse tumor tissues were fixed in 4% paraformaldehyde for 24 h before dehydration and paraffin embedding. Slice the tumor tissue at a thickness of 4 µm. After repairing the antigen in citrate buffer, the slices were incubated with 3% H_2_O_2_, washed with PBS, and then sealed in 10% goat serum. The cells were incubated overnight at 4 °C with the following antibodies: CD206 (1:800, 24595S, CST, USA) and F4/80 (1:500, NB600-404SS, Novus, USA). On the second day, after secondary antibody incubation, DAB staining and hematoxylin staining were performed, and the sections were observed under a microscope.

### Immunofluorescence (IHC) staining

Briefly, fresh mouse tumor tissues were quick-frozen embedded with OCT, and then the tumor tissue was sliced at a thickness of 6 µm. Next, the tissue sections were blocked with the prepared antibody blocking buffer for one hour. Then, diluted antibody was added [F4/80 (1:100, NB600-404SS, Novus, USA; CD206 (1:800, 24595S, CST, USA); MKP-1 (1:500, sc-373841, SANTA CRUZ, USA)] and incubated overnight at 4 °C. After the antibody was removed, the cells were washed with PBS three times, and fluorescently labeled antibody was added [goat anti-rat IgG (1:100, 112–545-003, Jackson, USA); goat anti-mouse IgG (1:100, 115–165-003, Jackson, USA)] and incubated for two hours at room temperature. The cell nuclei were stained with DAPI for 5 min after washing with PBS. The sections were observed under a confocal laser scanning microscope (OLYMPUS FV3000, Japan) (800 ×).

### Flow cytometry analyses

Tumor dissociation was performed using a Keygen tissue dissociation kit (KG829, Keygen Biotech, China) according to the manufacturer's instructions. Cells were sorted by utilizing CD45 MicroBeads (130–052-301, Miltenyi Biotec, Germany) and a MACS cell separation system (Miltenyi Biotec, Germany). Nonspecific staining through Fc receptor binding was blocked by incubation with 50 μl of rat anti-mouse CD16/CD32 (553,141, BD, USA). The following murine-specific flow cytometry antibodies were used: CD11b-APC (1:50, 130–113-231, Miltenyi Biotec, Germany), MHC class II-FITC (1:20, 130–102-168, Miltenyi Biotec, Germany), and CD206-PE (1:100, Clone: MR6F3, eBioscience, USA).

### RNA extraction and quantitative real-time PCR

Total RNA was extracted from the tumor tissues and cell lines using TRIzol Reagent (Takara, Tokyo, Japan) according to the manufacturer’s instructions. The mRNA quality was determined by A260/A280 (between 1.8 and 2.2) and A260/230 (> 1.7) ratios. Then, the RNA samples were reverse transcribed into cDNA using the PrimeScript™ RT reagent Kit (Takara, Tokyo, Japan), and real-time PCR was performed using TB Green® Premix Ex Taq™ II (Takara, Tokyo, Japan) according to the manufacturer's instructions. The primer sequences are shown in Table S1. The data were analyzed by the 2^−ΔΔCt^ method.

### Cell Counting Kit-8 proliferation assay

Cell proliferation was measured using a Cell Counting Kit-8 (CCK-8) (Dojindo Laboratories, Kumamoto, Japan) according to the manufacturer's instructions. The optical density was measured with a microplate reader (Bio-Rad, Hercules, CA, USA) at a wavelength of 450 nm.

### Wound healing assay

According to the experimental grouping, 2.0 × 10^6^ cells were placed on a 6-well plate, and the cell status was observed the next day. Three cell scratches perpendicular to the labeled horizontal line were made in parallel within each well using a 200 µl pipette tip. The cells were washed with PBS and then replaced with macrophage supernatant from different treatment groups. Each group of cells was placed under an inverted microscope. At least 3 fields of view were selected for each group to take photos. ImageJ software was used to calculate the change in scratch area for each group of cells at different time points (0 h, 24 h, 48 h) and calculate the wound healing rate: (initial scratch area -24 h/48 h scratch area)/initial scratch area × 100%.

### Transwell assay

THP-1 cells (1.0 × 10^6^) were inoculated into the lower Transwell chamber, induced according to the experimental groups, washed and cultured in 700 µl RPMI 1640 medium without FBS. A total of 5.0 × 10^4^ OSCC cells (PCI-37B, PCI-4B) were inoculated into the upper chamber of the Transwell chamber with 300 µl of RPMI 1640 medium without FBS. After coculture for 24 h, the membrane was fixed and stained. For each group, 3 fields of view were randomly selected for photography under a microscope, and the number of migrating cells in each group was counted using ImageJ software. For the invasion experiment, 100 μl of diluted Matrigel solution was evenly spread on the basement membrane of the Transwell upper chamber, and the other steps were the same as above.

### Statistical analysis

The statistical analysis was performed using R software (version 4.0.5) and GraphPad Prism (version 8.0.1). All experiments were repeated at least three times independently, and data are presented as the mean ± standard deviation (SD). The significance of differences between two groups was determined by Student’s t test. A *P value* < *0.05* was considered to indicate statistical significance.

## Results

### CCR7 deficiency decreases OSCC tumor burden

**Fig. 1:**
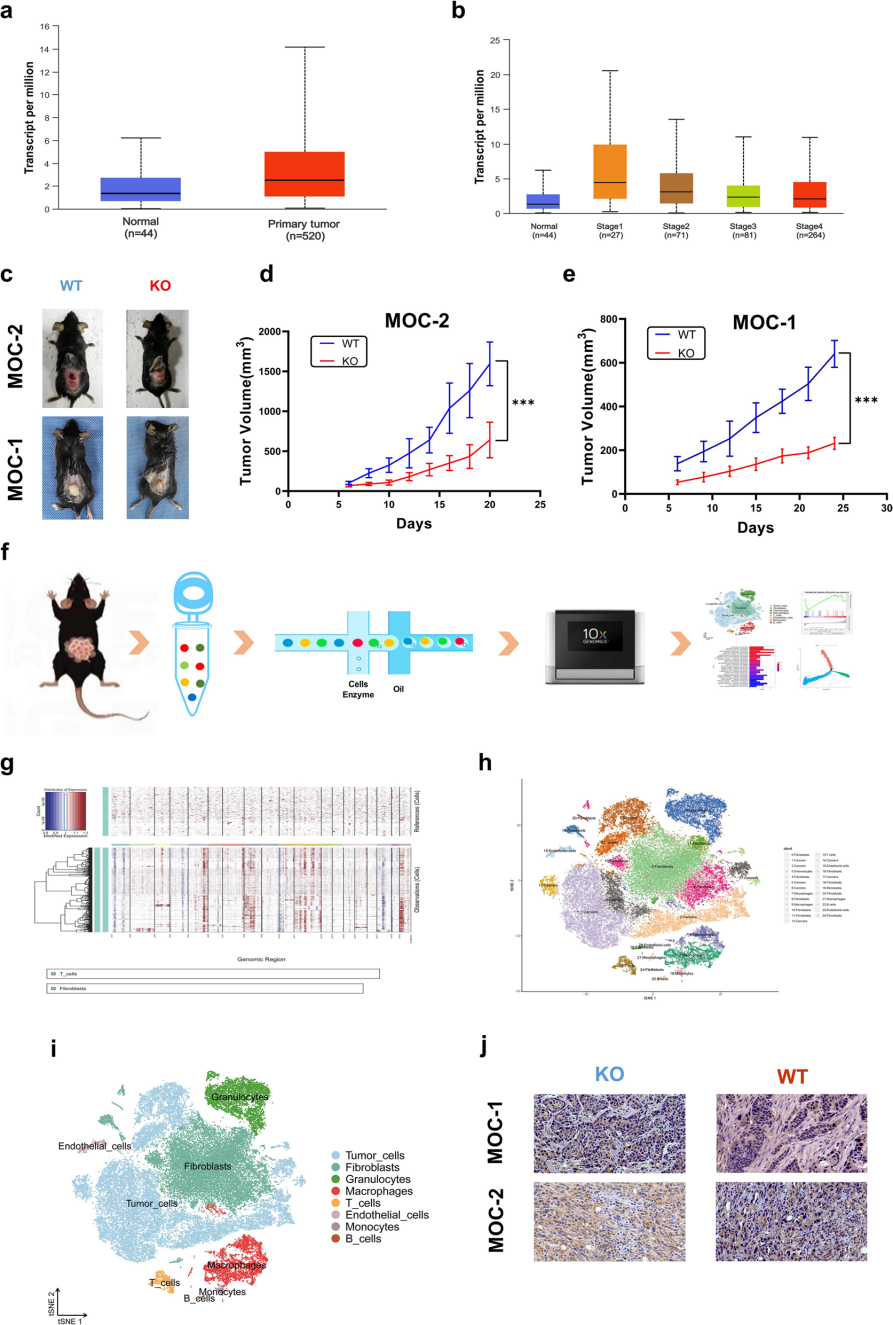
Subcutaneous MOC-1 and MOC-2 tumor growth are reduced in CCR7 deficient mice and scRNA-seq were carried out in KO and WT tumor tissues. **a** and **b** Expression level of CCR7 in HNSCC based on TCGA. **c** Subcutaneous tumor formation in CCR7 deficient (KO) and wild-type (WT) mice. **d** Compared to the WT tumor-bearing mice implanted with MOC-2 cells, the tumor growth in KO mice was significantly reduced. **e** Compared to the WT tumor-bearing mice implanted with MOC-1 cells, the tumor growth in KO mice was significantly reduced. (Student’s t-test, KO: *n* = 5, WT: *n* = 5, ***P < *0.001*). **f** Flow chart describes the scRNA-seq process. Tumor obtained from KO and WT mice are dissociated into single cells, captured in 10 × genomic platform for library construction and RNA sequencing. **g** InferCNV R pakage was used to distinguish benign and malignant cells. **h** 25 clusters were defined as specific cell types by Single R package and visualized by t-SNE. **i** Eight main cell types were identified by Seurat R package and visualized by t-SNE. **j** Immunohistochemistry shows the expression of CCR7 in tumor specimens of KO and WT group mice implanted with MOC-1 or MOC-2 cells

The expression level of CCR7 in head and neck squamous cell carcinoma (HNSCC) was higher than that in normal tissue (Fig. 1a) but differed between individual cancer stages according to the TCGA database (Fig. 1b). And it is consistent with our previous research results [32]. To clarify the role of CCR7 in the growth of OSCC, MOC-1 and MOC-2 cells were injected subcutaneously into the flanks of KO and WT mice. The results indicated that MOC-1 and MOC-2 OSCC cells were successfully engrafted into both KO and WT mice (Fig. 1c). Based on the tumor volume data, we found that the growth rate of OSCC in WT group mice was higher, and the tumor volume reached approximately 1500 mm^3^ (1593.86 ± 273.37) on day 20 implanted with MOC-2 cells and 600 mm^3^ (640.52 ± 55.32) on day 24 implanted with MOC-1 cells; however, the growth rate of OSCC in KO group mice was lower, and the tumor volume reached approximately 600 mm^3^ (640.46 ± 189.88) on day 20 implanted with MOC-2 cells and 200 mm^3^ (231.30 ± 24.67) on day 24 implanted with MOC-1 cells (Fig. 1d, e). These results indicated that CCR7 gene knockout can inhibit tumor growth and decrease the tumor burden of mice with OSCC. The immunohistochemical results for CCR7 indicate that CCR7 is expressed in tumor cells of the KO and WT groups, but in stroma cells, the expression level of CCR7 in the KO group was significantly lower than that in the WT group. (Fig. 1j).

### Single-cell RNA sequencing analysis

Because the tumor cells injected into the mice were the same, we analyzed the difference in tumor growth between the KO and WT groups caused by changes in the OSCC microenvironment. Then, we performed scRNA-seq on the tumors obtained from the WT groups (B2, B9, B47) and KO groups (B30, B76, B77) (Fig. 1f, Fig. S1a, b). A total of 70,316 cells were obtained after preliminary quality control. Then, the Doublet Finder R package was used to exclude the interference of multiple cells [33], and 58,836 cells without multiple cell interference were used for subsequent analysis (Fig. S2). Data reduction and cell clustering visualization were analyzed by the Seurat R package, and 25 cell clusters were visualized by t-SNE (Fig. S1c, d). The preliminary cell clustering by Single R [29, 34] can match some immune cells, but fibroblasts and tumor cells cannot be distinguished. Then, the inferCNV R package was used to distinguish benign and malignant cells according to reports [35–38] (Fig. 1g). Finally, eight clusters (1, 2, 5, 6, 8, 12, 14, and 17) were identified as malignant cells. The other 17 cell clusters were identified as benign cells, and then the final clustering annotation results were obtained (Fig. 1h). Eight main types of cells and their marker genes, including fibroblasts (Col1a2) [39, 40], macrophages (Arg1, Mrc1) [41], granulocytes (S100a8, S100a9) [42], T cells (Cd3g) [43], monocytes (Cd74, H2-eb1) [41, 44, 45], B cells (Cd79a, Cd79b) [46], endothelial cells (Cdh5, Pecam1) [47] and tumor cells, were finally identified (Fig. 1i). The proportion of each cell type among all cells in the KO and WT groups is shown in the supplementary materials (Table S2). Among these cells, monocytes were present in a higher proportion in the KO group than in the WT group (*P* = 0.046), but monocytes accounted for a small proportion of the total cells.

### Differential analysis of tumor cells

**Fig. 2:**
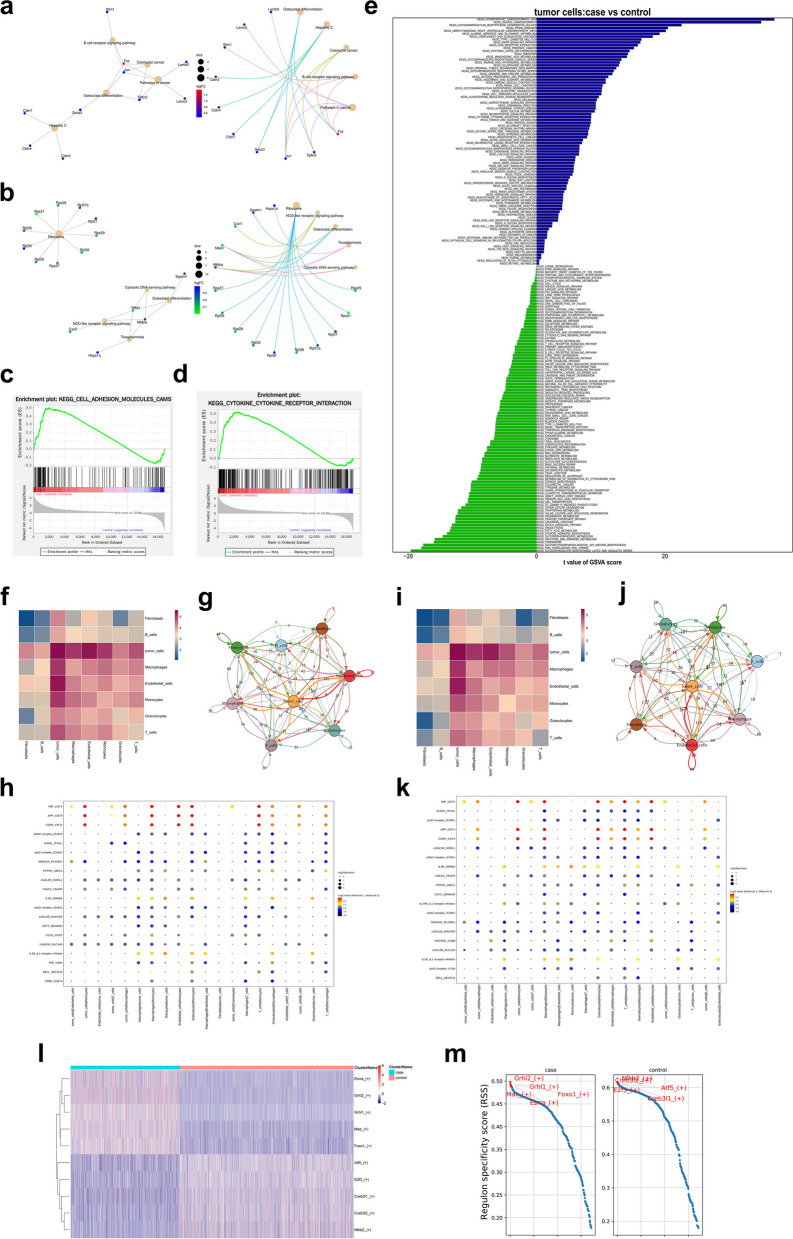
Heterogeneity analysis of tumor cells between KO and WT group. **a** KEGG result shows the enrichment of signal pathway based on positive top50 expressed gene in KO group. **b** KEGG result shows the enrichment of signal pathway based on negative top50 expressed gene in KO group. **c** and **d** GSEA analysis showed the regulation trend of signal pathway between KO and WT group. **e** GSVA analysis of KEGG between KO and WT group. **f** Heatmap of cell interactions in KO group analyzed by CellphoneDB. **g** The number of ligand–receptor pairs in KO group were shown in the net plot. **h** Top20 receptor-ligand pair of KO group was shown in heatmap. **i** Heatmap of cell interactions in WT group. **j** The number of ligand–receptor pairs in WT group were shown in the net plot. **k** Top20 receptor-ligand pair of KO group was shown in heatmap. **l** Top5 different transcription factors between KO and WT group were shown in heatmap. **m** Different Regulation specificity score (RSS) of the top5 transcription factors were shown between KO and WT group

As mentioned above, eight malignant cell clusters (1, 2, 5, 6, 8, 12, 14, and 17) were identified as tumor cells. The Find Clusters R package was used to analyze the different genes of the cell subset. Fig. S3a shows the top 20 differentially expressed genes in the KO group. Subsequent functional enrichment analysis was based on the top 50 upregulated and top 50 downregulated differentially expressed genes in the KO group. KEGG enrichment analysis showed that “MAPK signaling pathways”, “cell adhesion molecules (CAMs)” and “ECM-receptor interaction” were enriched in the genes that were upregulated in the KO group compared with the WT group, and these signaling pathways were closely related to the proliferation and metastasis of OSCC [48–50] (Fig. 2a); “ribosome”, “nod-like receptor signaling pathway” and “osteoclast differentiation” were enriched in genes downregulated in KO vs. WT tumor cells (Fig. 2b). GSEA results showed that “cell adhesion molecule (CAM)” and “cytokine‒cytokine receptor interaction signaling pathway” were enriched in genes upregulated between the KO group and the WT group (Fig. 2c, d, and the main enriched genes are shown in supplementary materials (Fig. S4a, b)). GSVA results showed that “alanine aspartate and glutamate metabolism”, “MAPK signaling pathway” and “ECM receptor interaction pathway” were enriched in the KO group, while “fructose and mannose metabolism”, “glycerophospholipid metabolism” and “fatty acid metabolism pathway” were enriched in the WT group (Fig. 2e). The functional enrichment analysis of tumor cells indicated that CCR7 gene knockout not only affected the enrichment of key signaling pathways in tumor cells but also affected the metabolic pathways of tumor cells.

To study the effect of CCR7 gene knockout on the interaction between various cells in OSCC, we performed cell communication analysis on various cells in the WT group and KO group via CellPhoneDB. We found that tumor cells had strong interactions with macrophages, endothelial cells, monocytes and T cells in both the KO and WT groups (Fig. 2f, i); however, the receptor‒ligand pairs between the KO and WT groups were different (Fig. 2g, j). Through CellPhoneDB analysis, we obtained the top 20 expressed receptor‒ligand pairs between different types of cells in the KO group (Fig. 2h) and WT group (Fig. 2k). SCENIC software was used to analyze the differences in transcription factor enrichment between the KO and WT groups as described previously [51], and the results showed that the top 5 transcription factors in the KO group were Grhl2, Grhl1, Maz, Foxo1 and Esrra; the top 5 transcription factors in the WT group were Nebfk2, Cr3bl2, E2f3, Atf5 and Creb3l1 (Fig. 2l, m).

**Fig. 3:**
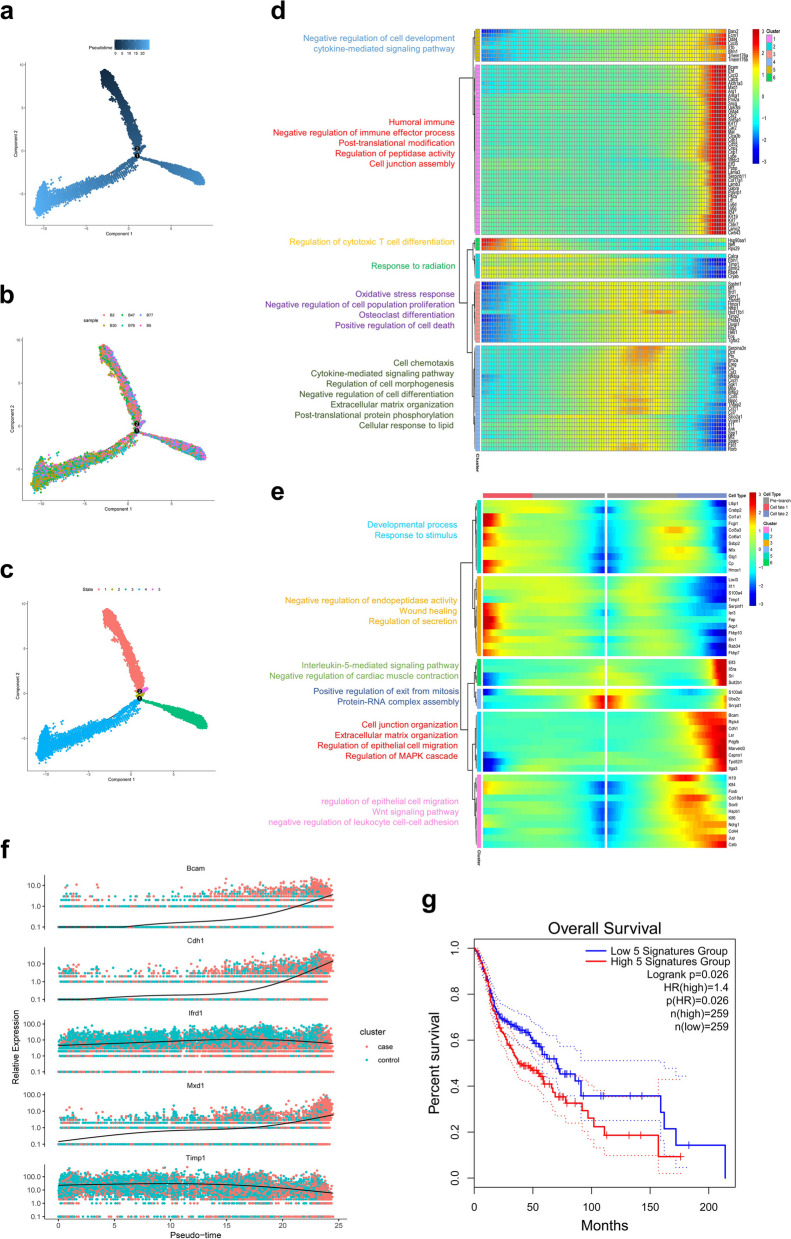
Trajectory analysis of tumor cells. **a** Trajectory analysis diagrams of pseudotime was shown. **b** The pseudotime in different samples. **c** The pseudotime in different states. **d** The top100 differential gene function enrichment heatmap. **e** The differential expression genes of different branches and GO BP pathways of different clusters were shown in heatmap. **f** The Dynamic changes of top5 differential expression genes determined cell fate between KO and WT group were shown. **g** Survival analysis of OSCC gene features using TCGA-PAAD based on Fig. 3f on the Gepia2 website (http:// gepia2. cancer- pku. cn/# index)

To further dissect the mechanisms of tumor progression, trajectory analysis for tumor clusters was carried out using the Monocle 2 R package. Trajectory analysis diagrams were obtained in different clusters, different states and different samples according to the pseudotime (Fig. 3a-c). We selected the top 100 genes with the most significant differences and similar trends during the pseudotime process for clustering visualization. Figure 3d shows the GO functional enrichment in six clusters. Next, we conducted BEAM analysis on node one, which had a significant impact on cell trajectory. The results indicated that genes highly expressed in the pre-branch node were mainly enriched in “positive regulation of exit from mitosis” and “Protein‒RNA complex assembly”; genes enriched in “response to stimulus”, “negative regulation of endopeptidase activity”, “Wound healing” and “regulation of secretion” were highly expressed in the cell fate1 node; genes enriched in “cell junction organization”, “Extracellular matrix organization”, “regulation of epithelial cell migration” and “regulation of MAPK cascade” were highly expressed in the cell fate2 node (Fig. 3e). Dynamic changes in the top 5 representative genes (Bcam, Cdh1, Ifrd1, Mxd1, and Timp1) that determine the fate of tumor cells during the pseudotime process are shown in Fig. 3f. The GEPIA2 analysis results indicate that the top 5 key genes (Bcam, Cdh1, Ifrd1, Mxd1, and Timp1) based on the trajectory analysis of tumor cells can affect the survival of tumor patients (Fig. 3g).

### Heterogeneity analysis of seven stromal cell types

As mentioned above, the marker gene expression of endothelial cells, monocytes, B cells, granulocytes, T cells, fibroblasts and macrophages is shown in the supplementary materials (Fig. S5a-g, S6a-g). The top 20 differentially expressed genes in these seven stromal cell types in the KO group are shown in Fig. S3b-h. Subsequent functional enrichment analysis was based on the top 50 upregulated and top 50 downregulated differentially expressed genes in the KO group. The KEGG results showed the positively enriched signaling pathways in the KO group and the negatively enriched pathways in the WT group in endothelial cells (Fig. S7a), monocytes (Fig. S7b), B cells (Fig. S7c), granulocytes (Fig. S7d), T cells (Fig. S7e), fibroblasts (Fig. S7f) and macrophages (Fig. S7g). The GSVA results showed different enriched signaling pathways between the KO and WT groups in endothelial cells (Fig. S8), monocytes (Fig. S9), B cells (Fig. S10), granulocytes (Fig. S11), T cells (Fig. S12), fibroblasts (Fig. S13) and macrophages (Fig. S14). The GSEA results were as follows: in endothelial cells, genes in the “cell-cycle signaling pathway” were positively enriched in the KO and WT groups, and genes in the “VEGF signaling pathway” were negatively enriched in the KO and WT groups (Fig. S15a-d); in monocytes, the “Toll-like signaling pathway” and “nod-like signaling pathway” were positively enriched in the WT and KO groups (Fig. S16a-d); in B cells, “cytokine‒cytokine receptor interaction” and “ECM receptor interaction signaling pathway” were positively enriched in the KO but negatively enriched in the WT group (Fig. S17a-d); in granulocytes, the “nod-like receptor signaling pathway” and “Toll-like receptor signaling pathway” was positively enriched in the KO and WT groups (Fig. S18a-d); in T cells, the “chemokine signaling pathway” and “JAK-STAT signaling pathway” were positively enriched in the KO group and negatively enriched in the WT group (Fig. S19a-d); in fibroblasts, the “TGF-β signaling pathway” and “MAPK signaling pathway” were positively enriched in the KO group vs. the WT group (Fig. S20a-d); in macrophages, the “TGF-β signaling pathway” were negatively enriched in KO and WT groups and “chemokine receptor signaling pathway” were positively enriched in the KO group, but negatively enriched in the WT group (Fig. S21a-d).

We subsequently performed trajectory analysis in T cells, granulocytes, and macrophages, which accounted for a significant proportion of inflammatory cells based on scRNA-seq results. We obtained trajectory diagrams for different clusters, different samples and different states during pseudotime in T cells (Fig. S22a-c), granulocytes (Fig. S23a-c) and macrophages (Fig. S24a-c). As mentioned above, the top 100 genes with the most significant differences and similar trends during pseudotime were selected and divided into six clusters. For T cells, GO enrichment analysis results for six clusters are shown in Fig. S22d. The BEAM analysis results on node one indicated that “cell killing”, “negative regulation of viral transcription”, “humoral immune response”, and “negative regulation of angiogenesis” were enriched in the pre-branch node; “positive regulation of leukocyte activation”, “cellular process”, “leukocyte aggregation”, and “regulation of prostaglandin biosynthetic process” were enriched in the cell fate 2 node; and “regulation of inflammatory response”, “regulation of macrophage activation”, and “regulation of leukocyte activation” were enriched in the cell fate 1 node (Fig. S22e). Dynamic changes in the top 5 representative genes (Ccl5, Gzma, Gzmc, Gzd, Gzmf) that determine the fate of T cells during the pseudotime process are shown in Fig. S22f. In granulocytes, GO enrichment results in six clusters are shown in Fig. S23d. BEAM analysis results on node one indicated that “Cytokine production involved in immune response”, “Eosinophil chemotaxis”, and “regulation of TRAIL-activated apoptotic signaling pathway” were enriched in prebranch; “NF-κB signaling pathway”, “positive regulation of leukocyte migration”, “negative regulation of cell cycle G1/S phase transition”, and “negative regulation of cysteine-type endopeptidase activity” were enriched in the cell fate 2 node; and “MAPK signaling pathway”, “positive regulation of angiogenesis” and “cellular response to hypoxia” were enriched in the cell fate 1 node (Fig. S23e). Dynamic changes in the top 5 representative genes (Ccl3, Ccl4, Ccl6, Hk2, Slpi) that determine the fate of granulocytes during pseudotime are shown in Fig. S23f. For macrophages, Fig. S24d shows the GO enrichment result in six clusters. The BEAM analysis results on node one indicated that “neutrophil chemotaxis”, “positive regulation of MAPK cascade”, “negative regulation of catalytic activity”, and “negative regulation of metallopeptidase activity” were enriched in the pre-branch node; “tissue remodeling”, “extracellular matrix organization” and “skeletal system development” were enriched in cell fate 1; and “JAK-STAT signaling pathway”, “positive regulation of leukocyte activation”, and “positive regulation of tumor necrosis factor production” were enriched in the cell fate 2 node (Fig. S24e). The dynamic changes in the top 5 representative genes (Acp5, Cd52, Ckb, S100a4, S100a6) that determine the fate of macrophages during the pseudotime process are shown in Fig. S24f, and GEPIA2 analysis showed that changes in the top 5 key genes can affect the survival time of tumor patients (Fig. S24g). Next, we performed reclustering analysis on T cells and granulocytes. We obtained six cell subpopulations in T cells (Fig. S22g) and seven cell subpopulations in granulocytes (Fig. S23g). The cell number, gene number and UMI number of six T-cell subpopulations and seven granulocyte subpopulations are shown in Fig. S22h and Fig. S23h.

### CCR7 knockout inhibits M2 macrophage polarization, which suppresses the biological process of OSCC

**Fig. 4:**
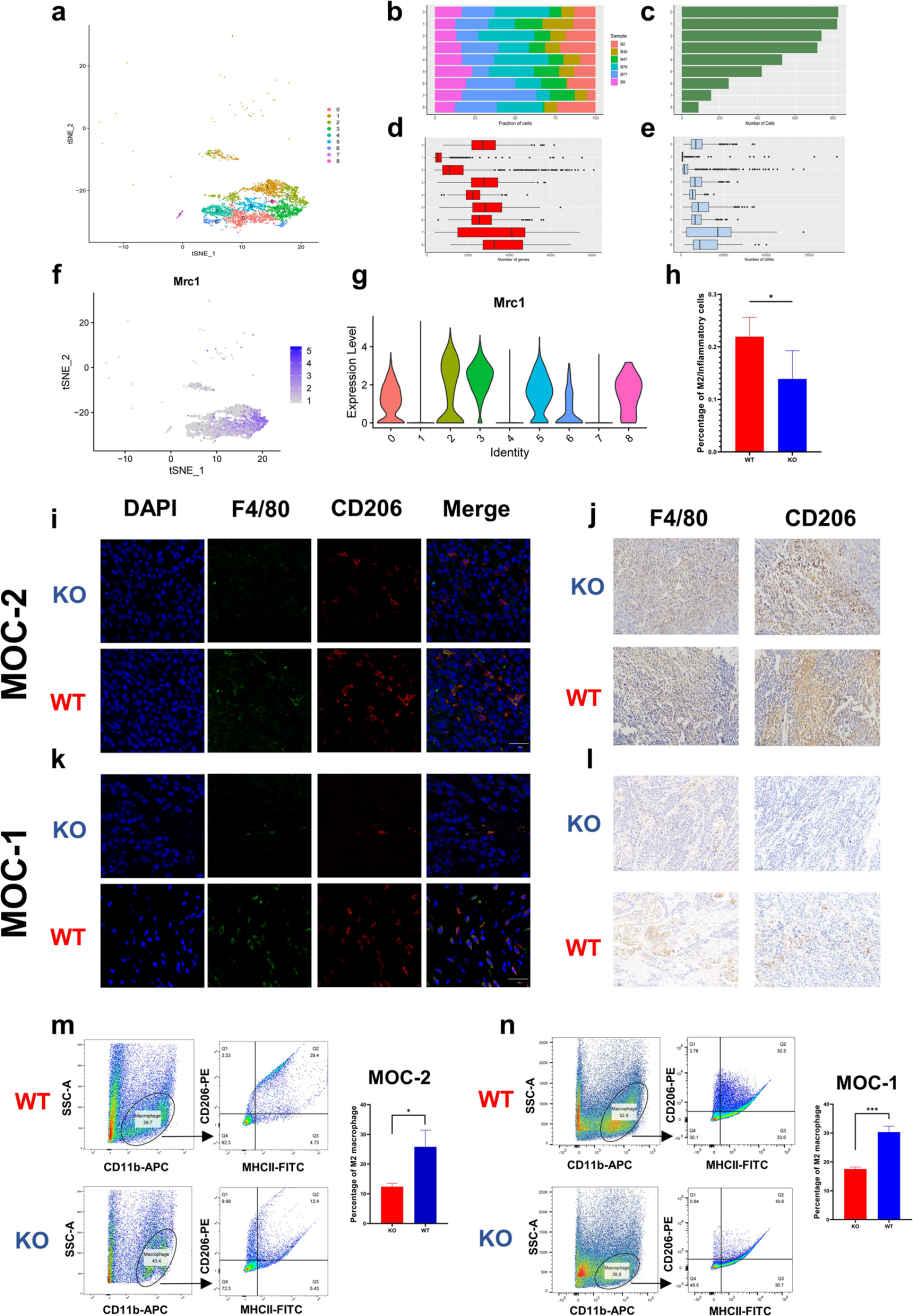
Macrophage subpopulation analysis. **a** Subpopulation of macrophages were shown by t-SNE. **b** Fraction of cells in different clusters among six samples. **c** Number of cells in different clusters. **d** Number of genes in different clusters. **e** Number of UMIs in different clusters. **f** and **g** The expression level of Mrc1 in different subclusters of macrophage. **h** Proportion of M2 macrophages between KO and WT group in scRNA-seq results. **i** Immunofluorescence staining shows the expression of M2 macrophage between KO and WT group implanted with MOC-2 cells. **j** Immunohistochemistry demonstrated the expression of M2 macrophage between KO and WT group implanted with MOC-2 cells. **k** Immunofluorescence staining shows the expression of M2 macrophage between KO and WT group implanted with MOC-1 cells. **l** Immunohistochemistry demonstrated the expression of M2 macrophage between KO and WT group implanted with MOC-1 cells. **m** Flow cytometry analysis showed the expression levels of M2 macrophages between KO and WT group implanted with MOC-2 cells. **n** Flow cytometry analysis showed the expression levels of M2 macrophages between KO and WT group implanted with MOC-1 cells. (Student’s t-test, KO: *n* = 3, WT: *n* = 3, *P < *0.05*.)

We performed reclustering analysis on macrophages in the KO group and WT group and obtained 9 clusters (Fig. 4a). The number of genes, cells and UMI in each cluster are shown in Fig. 4b-e. According to the marker gene expression of CD206 (also named Mrc1), clusters 2, 3 and 5 were defined as M2 macrophages (Fig. 4f, g). ScRNA-seq results showed that the proportion of M2 macrophages in inflammatory cells in the KO group was lower than that in the WT group (*P* < 0.05, Fig. 4h). Immunohistochemical analysis and immunofluorescence staining both demonstrated that there were fewer M2 macrophages (F4/80, CD206) in KO tumor tissue than in WT tumor tissue implanted with MOC-1 or MOC-2 cells (Fig. 4i-l). Flow cytometry analysis showed that the proportion of M2 cells/inflammatory cells in the KO group was significantly lower than that in the WT group (Fig. 4m, n). These results indicated that CCR7 gene knockout can reduce the infiltration of M2 macrophages in OSCC.

**Fig. 5:**
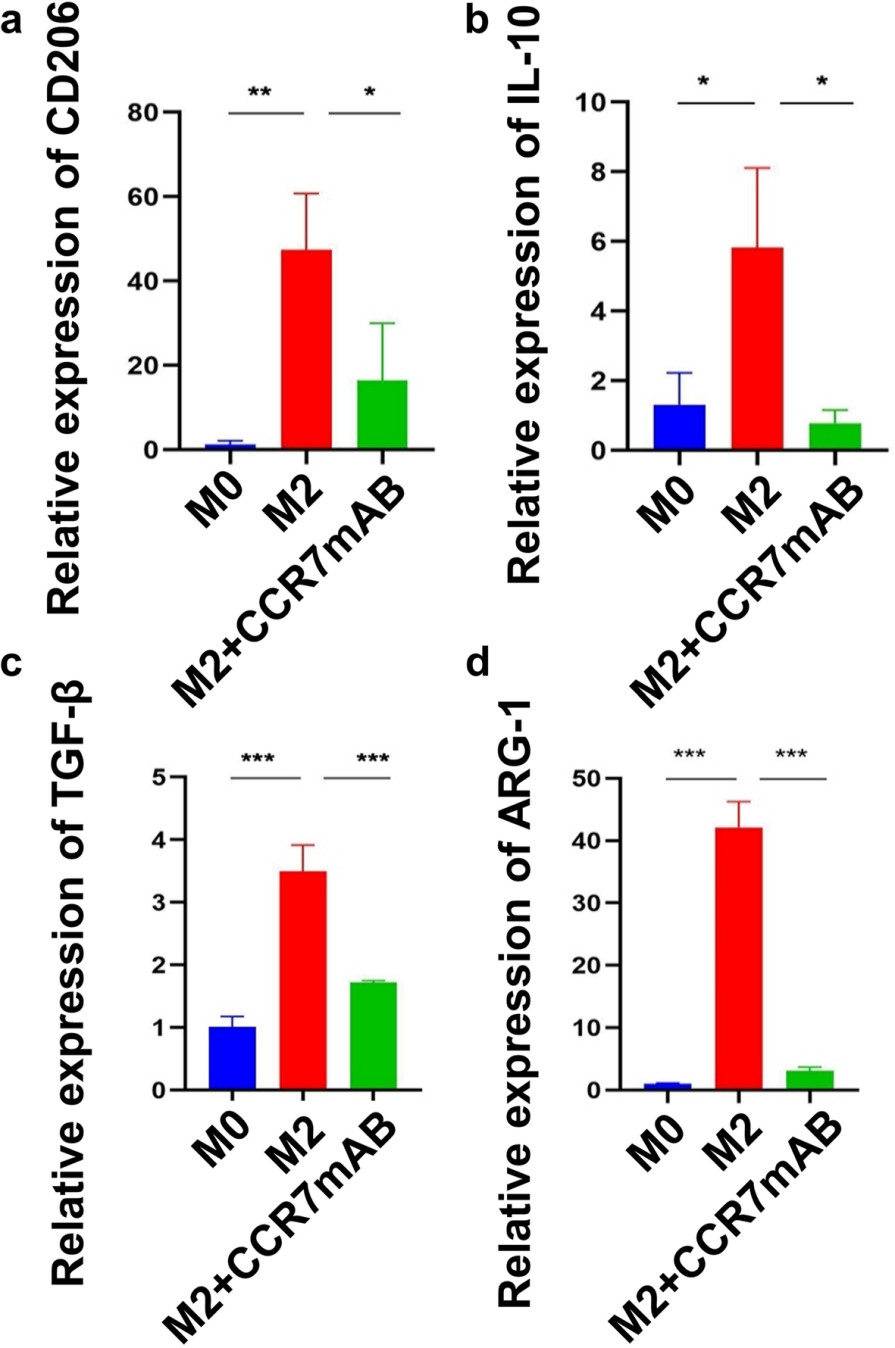
M2 marker gene expression level. **a** CD206 expression level in different groups. **b** IL-10 expression level in different groups. **c** TGF-β expression level in different groups. **d** ARG-1 expression level in different groups

**Fig. 6:**
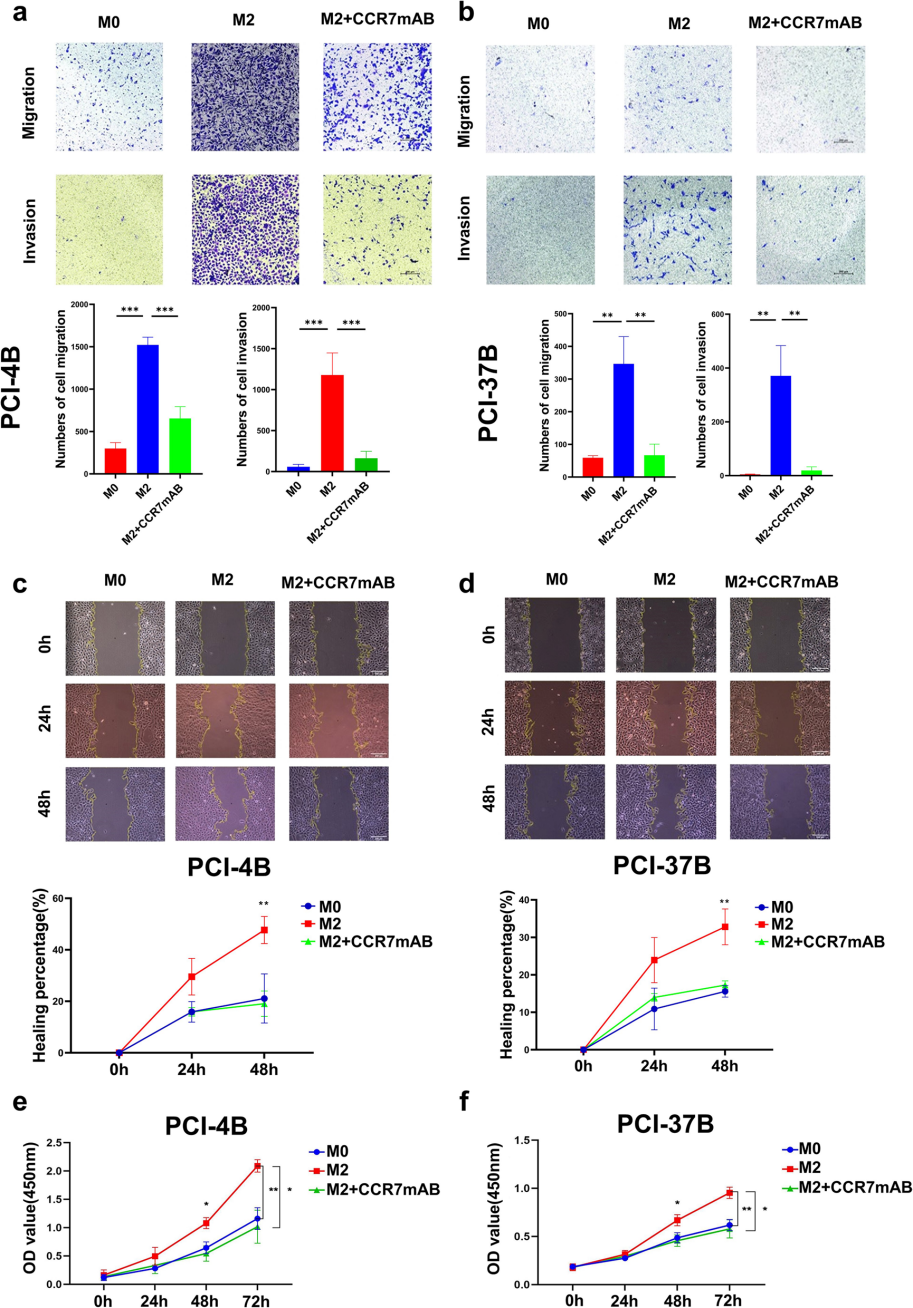
CCR7 knockout inhibits M2 macrophage polarization. **a** and **b** Transwell assay shows the invasion and migration ability of PCI-37B and PCI-4B cells between different experimental groups. **c** and **d** Wound healing assay shows the migration ability of PCI-37B and PCI-4B cells between different experimental groups. **e** and **f** CCK8 assay shows the proliferation ability of PCI-37B and PCI-4B cells between different experimental groups. (One-way analysis of variance, *P < 0.05, **P < 0.01, ***P < *0.001*.)

In the in vitro experiment, THP-1 cells were induced into M0 macrophages by PMA and then induced into M2 macrophages by IL-4 + IL-13 as described previously. The real-time PCR results showed that the expression levels of the M2 markers CD206, ARG-1, IL-10 and TGF-β in the M2 group were higher than those in the M0 group, indicating induction success (Fig. 5a-d). This induction was significantly inhibited after the addition of CCR7 mAb, indicating that CCR7 can block M2 polarization (Fig. 5a-d). The abilities of cell migration and invasion were increased significantly when PCI-4B and PCI-37B cells were co-cultured with M2 macrophages; however, when we pretreated M2 macrophages with CCR7 mAb, the migration and invasion abilities of tumor cells (PCI-4B, PCI-37B) were significantly decreased (Fig. 6a, b). Similar results were observed in the wound healing assay and CCK-8 assay, indicating that CCR7-induced M2 polarization can promote tumor cell survival, migration and invasion (Fig. 6c-f).

**Fig. 7:**
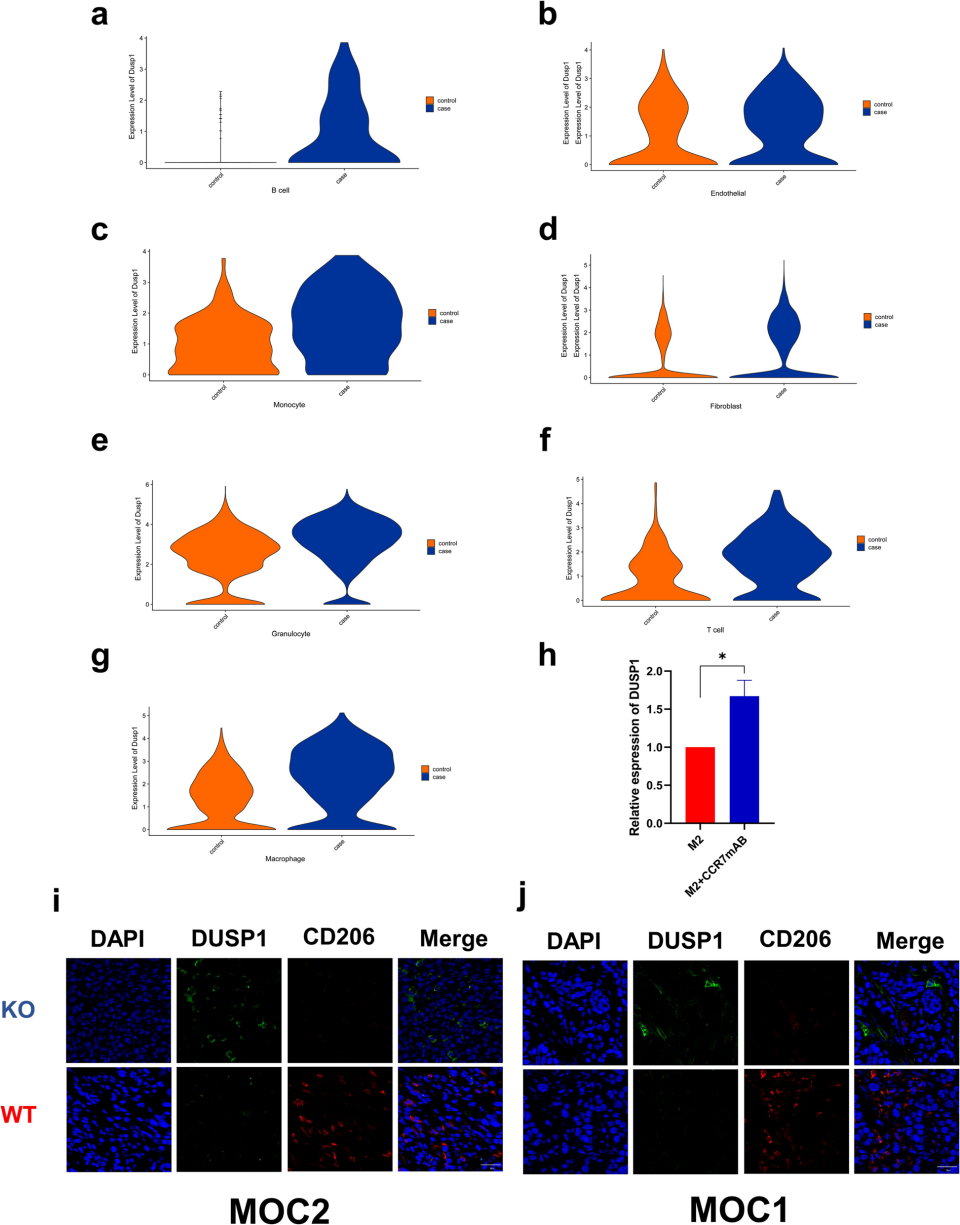
Expression levels of Dusp1 in different types of cells between KO and WT group. **a**-**g** Violin plots show that DUSP1 expression levels in KO group were higher than WT group in seven stromal cells. Case represents KO group, control represents WT group. **h** The expression levels of Dusp1 by RT-PCR (Student’s t-test, M2: *n* = 3, M2 + CCR7 mAB: *n* = 3, *P < *0.05*.). **i** Immunofluorescence staining shows the expression of Dusp1 in M2 macrophage between KO and WT group implanted with MOC-2 cells. **j** Immunofluorescence staining shows the expression of Dusp1 in M2 macrophage between KO and WT group implanted with MOC-1 cells

To study the mechanism of CCR7, we compared the gene changes in different cells between the KO and WT groups according to the scRNA-seq results. We found that in all stromal cells, including B cells, T cells, endothelial cells, granulocytes, macrophages, monocytes and fibroblasts, CCR7 knockout generally induced high *Dusp1* mRNA expression (Fig. 7a-g). In an in vitro experiment, the real-time PCR results showed that CCR7 mAb can promote *Dusp1* expression in the M2 macrophages we induced (Fig. 7h). Immunofluorescence staining also showed that MKP-1 (encoded by *Dusp1* mRNA) was largely located in M2 macrophages and was highly expressed in the CCR7 knockout group (Fig. 7i, j). Since our previous studies demonstrated that *Dusp1* gene deficiency can promote the growth of OSCC and enhance M2 macrophage polarization [52], we suggest that CCR7 promotion of M2 polarization is dependent on *Dusp1* loss.

## Discussion

Studies have shown that elevated CCR7 expression levels are associated with lymph node metastasis in a variety of tumors (including breast cancer [53, 54], esophageal cancer [55], cervical cancer [56], thyroid cancer [57], lung cancer [58] and oral squamous cell carcinoma [59]). In OSCC, a high expression level of CCR7 is associated with a poor prognosis [18, 60] and CCR7-induced activation of NF-κB through the PI3K/Akt/mTOR signaling pathway is critical for OSCC cell survival and prognosis [32]. In addition, the expression levels of CCR7 and integrin αvβ3 are positively correlated with the tumor size, clinical stage and lymph node metastasis of OSCC, and cell adhesion and migration are promoted by inducing integrin αvβ3 phosphorylation [61]. Further, CCR7 can activate the MAPK signaling pathway by stimulating the phosphorylation of ERK1/2 and JNK, thus promoting the proliferation, invasion, and migration of OSCC cells [62]. Other data indicates that noncoding RNAs (such as miR-1275 and let-7e-5p) affect the biological function of OSCC by regulating the expression of CCR7 [63, 64]. Collectively, these studies indicate that CCR7 can promote the proliferation, migration and invasion of OSCC cells through a variety of non-redundant cellular mechanisms. However, relatively less information is known about mechanisms used by CCR7 on tumor infiltrating immune cells that would impact the TME and contribute towards tumor progression. In this research, we found that CCR7 gene knockout can significantly inhibit tumor growth and altered the microenvironment especially reducing the infiltration of M2 macrophages of OSCC, CCR7 may promote M2 macrophage polarization by inhibiting *Dusp1* expression, thus promoting the proliferation and metastasis of OSCC.

To study the tumor immune microenvironment, an immune competent mouse model is essential. As mentioned above, Boyle et al. generated a bigenic mouse model of breast cancer combined with CCR7 deletion and revealed that CCR7 ablation results in a considerable delay in tumor onset as well as a significantly reduced tumor burden [23]. The results first demonstrated the role of CCR7 in immune mouse cancer in vivo, but the authors just focused on cancer stem like-cells and did not explore the TME alterations. In this work, we constructed an OSCC mouse model and found that CCR7 knockout significantly inhibited OSCC growth compared with that in the WT group. The result is consistent with that of Boyle et al. in breast cancer, with one difference: the model we constructed is an allograft model, which means that the tumor cells we injected into WT and KO mice were the same in terms of number and characteristics. Therefore, we speculate that CCR7 exerts its effect in OSCC in our study by changing the tumor microenvironment.

The tumor microenvironment is a dynamic and complex changing system, and it is difficult to accurately obtain the specific mechanism by which the TME affects tumor development. The scRNA-seq results from fifteen primary nasopharyngeal carcinoma tumors (NPCs) and one normal sample demonstrated that the signatures of macrophages, plasmacytoid dendritic cells (pDCs), CLEC9A + DCs, natural killer (NK) cells, and plasma cells were significantly associated with improved survival outcomes in NPC [65]. Through scRNA-seq analysis of human lung cancer tissues, 52 stromal cell subtypes were identified, and the effect of marker genes on the prognosis of lung cancer was determined [66]. Cell trajectory analysis showed that multiple tumor-related pathways and transcription factors were differentially expressed during the progression of pancreatic ductal adenocarcinoma [67]. An increasing number of studies have indicated that scRNA-seq analysis is an ideal method for TME research, but little related research has been done in OSCC. To investigate which stromal cells are affected by CCR7 in OSCC, we performed scRNA-seq analysis in WT and KO tumor tissues. The results showed that there were differences in monocytes between the KO group and WT group; however, the proportion of monocytes among the total cells was too small, so the role of these cells may be very limited.

TAMs are the most abundant tumor-infiltrating immune cells in OSCC [68]. High levels of TAMs in the TME have been shown to be associated with lymph node metastasis and advanced disease stage in OSCC [69, 70]. Generally, TAMs can be divided into two subsets: immunostimulatory macrophages (M1 type macrophages) and immunomodulatory macrophages (M2 type macrophages). M1 macrophages secrete γ interferon (IFN-γ) and other inflammatory cytokines, whereas M2 macrophages produce immunosuppressive cytokines, such as interleukin 10 (IL-10), which are involved in tumor immune escape in the TME and promote tumor cell proliferation [71, 72]. M1-like TAMs are encapsulated in the internal region of the tumor mass, while M2-like TAMs are enriched in the peripheral region of the tumor, which suggests that M2-like TAMs play an immunosuppressive role in the TME and assist in tumor invasion [73]. In this study, although there was no difference in the proportion of macrophages among total cells, the proportion of M2 macrophages among inflammatory cells in the KO group was significantly lower than that in the WT group based on our scRNA-seq results. Consistent with scRNA-seq data, immunohistochemical, immunofluorescence staining and flow cytometry analyses also demonstrated that M2 macrophages were decreased in CCR7 knockout tissues. Therefore, additional studies focused on M2 macrophages. Subsequently, we found that CCR7 can promote OSCC cell growth, migration, and invasion by polarizing M2 macrophages. This is an interesting finding because some other investigators consider CCR7 as a marker gene of M1 macrophages [74–76]. Indeed, some research has demonstrated that CCR7 expression is unchanged in the human monocyte lines THP-1 and U937 and in primary monocyte-induced M1 macrophages but increases in the cytoplasm in human primary CD14^+^ mononuclear cell-induced M2 macrophages [77, 78]. This may be due to different research objects, immune microenvironments, and cell type definitions. Therefore, CCR7’s role in macrophages is indeed quite complex that requires additional exploration to fully understand.

In this work, scRNA-seq results indicated that the expression level of *Dusp1* in the KO group was significantly higher than that in the WT group in monocytes, endothelial cells, B cells, T cells, macrophages, granulocytes and fibroblasts. Dual-specificity phosphatase-1 (*Dusp1*, encoding for MKP-1), initially found in cultured mouse cells, is generally thought to be a MAPK family inhibitor [79]. MAPK family members include extracellular signal-regulated protein kinases (ERKs), JNKs and p38 MAPKs, which play important roles in cell proliferation and apoptosis. In general, the ERK1/2 cascade seems to mediate signals that promote cell proliferation, differentiation, or survival, while the JNK and p38 MAPK cascades seem to be involved in cell responses to stress [80]. Research has shown that *Dusp1* can inactivate ERK, JNK and p38 in vivo through dephosphorylation [44, 81–83]. *Dusp1* can negatively regulate the immune response by directly dephosphorylating p38 and JNK and may also compete with upstream mapkks and downstream substrates to participate in the regulation of MAPKs by binding with p38 or JNK [84]. Our previous research has shown that *Dusp1* gene deficiency can promote the polarization of M2 macrophages and the growth of OSCC in mice [85]. According to previous results and the results of this study, in which CCR7 knockout inhibited M2 macrophage polarization and promoted *Dusp1* expression in M2 macrophages, we can conclude that CCR7 promotes OSCC growth via *Dusp1*-regulated M2 macrophage polarization. Further research is needed to confirm the regulatory mechanism between CCR7 and *Dusp1* and their impact on the tumor microenvironment of oral squamous cell carcinoma.

This work has some limitations. Firstly, only three wild-type (WT) and three knockout (KO) mice data were performed scRNA-seq. Although it fulfills the sample requirements for statistical analysis of inter-group differences, the risk of bias is concerned. Therefore, we furtherly carried out a series of in vitro experiments to verify the results of single-cell sequencing analysis. In subsequent research, we will increase the sample size for extensive validation. Secondly, mouse models may not fully represent the human OSCC microenvironment. Although mouse model is economic, available and widely used, with 90% of the genes highly similar to human genes, it still can’t perfectly mirror the human OSCC microenvironment. The experiment on human being is needed to follow up in subsequent study. Finally, knockout mice may have off-target effects or compensation mechanisms, thereby affecting the results of the study. To settle this matter, CCR7 knock-out mice were crossed with inbred C57BL6 mice for more than three generations to avoid potential off-target changes caused by CRISPR-Cas9 genome editing and many in vitro experiment were performed to demonstrate the effect of CCR7 on OSCC microenvironment.

## Conclusion

The above results indicate that CCR7 gene knockout can significantly inhibit tumor growth and affect the TME in OSCC. ScRNA-seq and in vitro experimental results indicate that CCR7 may promote M2 macrophage polarization by inhibiting *Dusp1* expression, thus promoting the proliferation and metastasis of OSCC.

### Supplementary Information

**Supplementary Material 1.**

## Data Availability

The datasets used and/or analyzed during the current study are available from the corresponding author on reasonable request. All data generated or analyzed during this study are available in this article [and its supplementary files] and in public repositories.

## Abbreviations

OSCC: Oral squamous cell carcinoma
CCR7: CC Chemokine receptor 7
TME: Tumor microenvironment
scRNA-seq: Single-cell RNA sequencing
TNBC: Triple-negative breast cancer
PD-L1: Programmed death ligand-1
PD-1: Programmed death-1
FDA: Food and Drug Administration
TAM: Tumor-associated macrophage
CAF: Tumor-associated fibroblast
