# Supplementary material for: Analysis of the effect of CCR7 on the microenvironment of mouse oral squamous cell carcinoma by single-cell RNA sequencing technology
Source: J Exp Clin Cancer Res. 2024 Mar 27;43:94. doi: 10.1186/s13046-024-03013-y (PMC10976828; doi:10.1186/s13046-024-03013-y)
Supplement: Supplementary file 1 — Supplementary Material 1. [file 13046_2024_3013_MOESM1_ESM.docx]

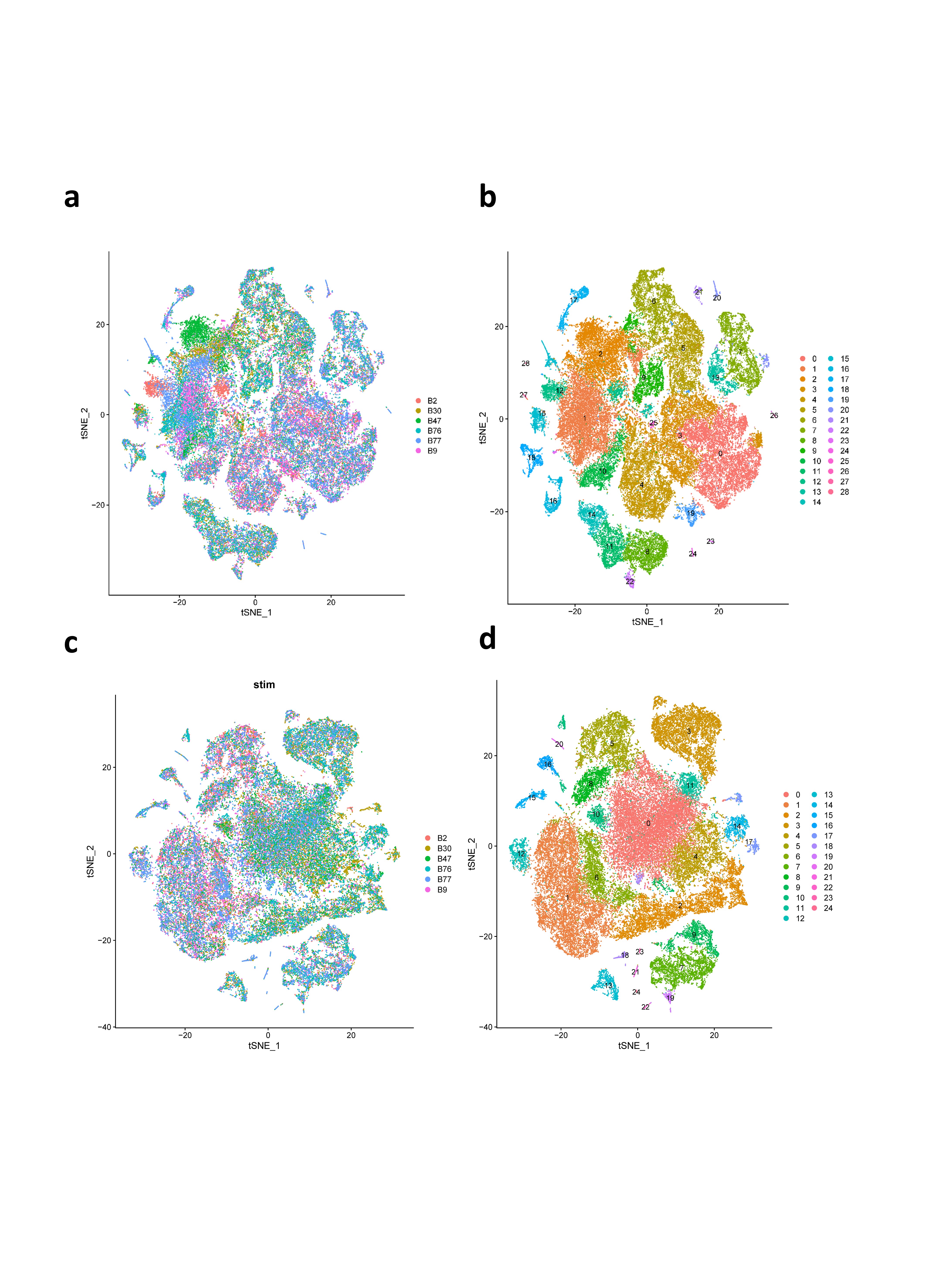


**Figure S1.** Tumor scRNA-seq with preliminary quality control. a. Cell clusters of six samples were visualized by t-SNE without removing multiple cells. B2, B9 and B30 represent WT group mice; B30, B76 and B77 represent KO group mice. b. 29 cell clusters of six samples were visualized by t-SNE without removing multiple cells. c. Cell clusters of six samples were visualized by t-SNE. B2, B9 and B30 represent WT group mice; B30, B76 and B77 represent KO group mice. d. 25 cell clusters of six samples were visualized by t-SNE

**
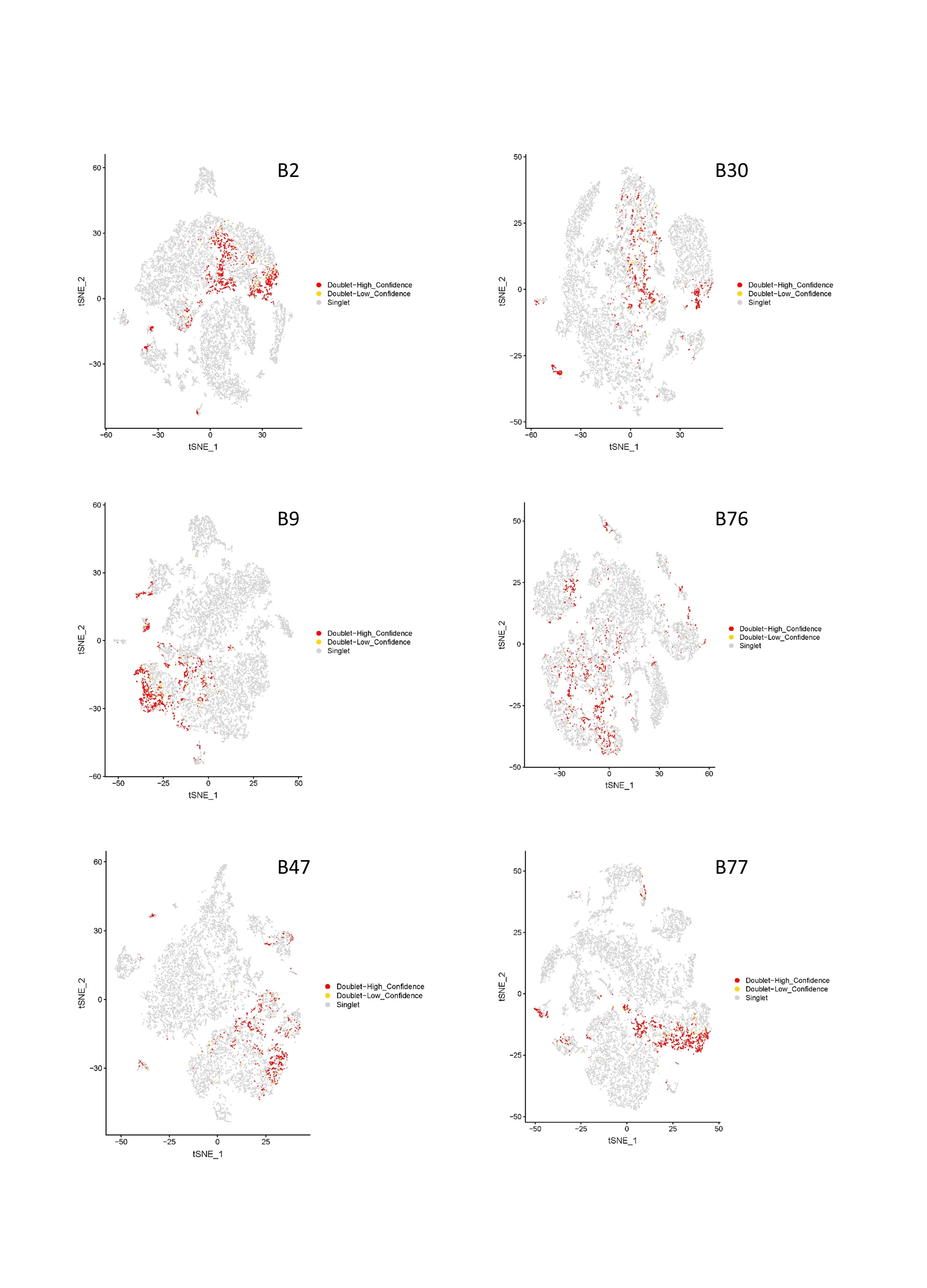
**

**Figure S2.** Excluding the interference of multiple cells by Doublet Finder R package. B2, B9 and B30 represent WT group mice; B30, B76 and B77 represent KO group mice.

**
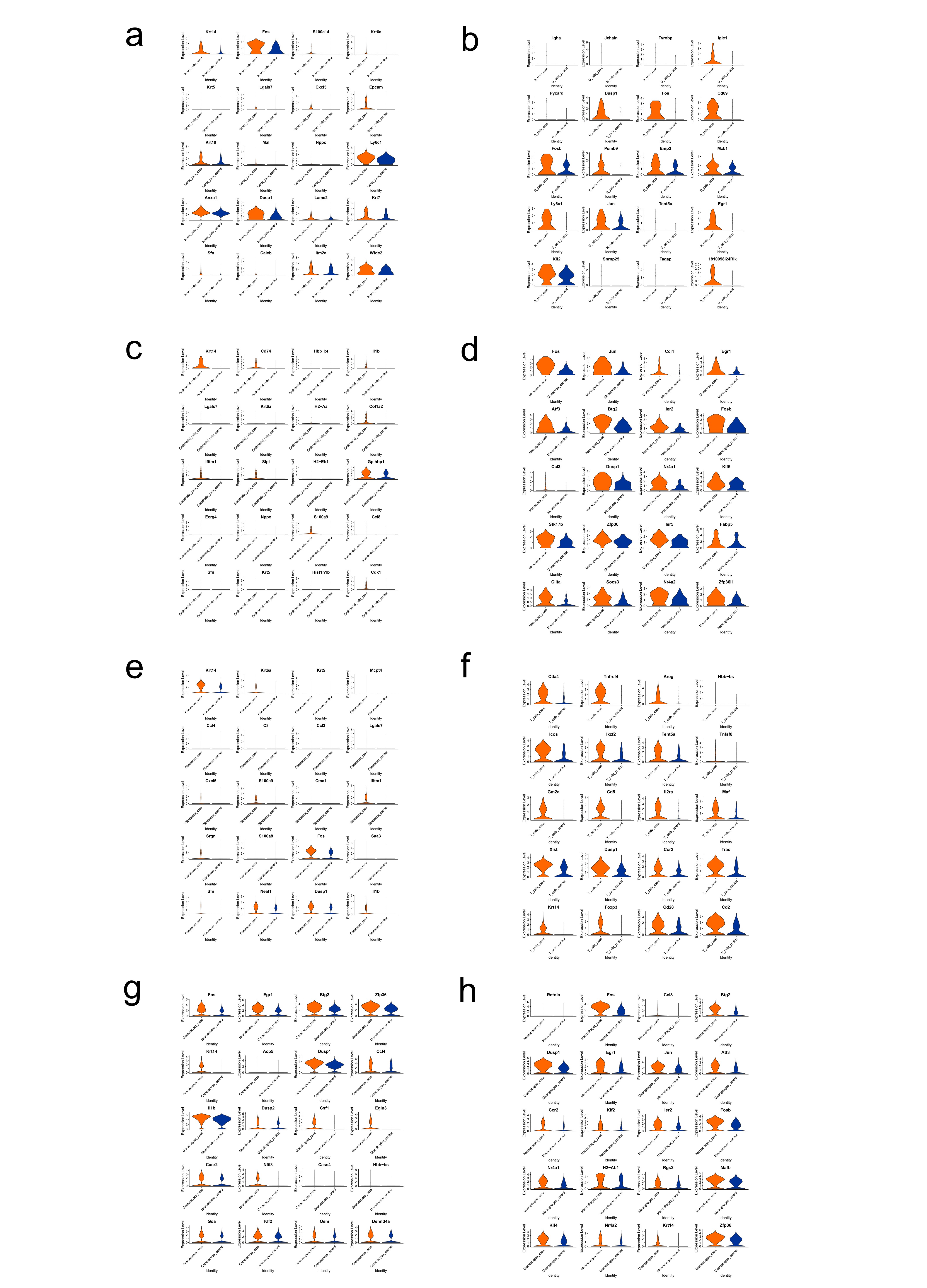
**

**Figure S3.** Violin plots of top20 gene expression levels in KO group. a. Tumor cells top20 gene expression levels. b. B cells top20 gene expression levels. c. Endothelial cells top20 gene expression levels. d. Monocytes top20 gene expression levels. e. Fibroblasts top20 gene expression levels. f. T cells top20 gene expression levels. g. Granulocytes top20 gene expression levels. h. Macrophages top20 gene expression levels. case represents KO group; control represents WT group.

**
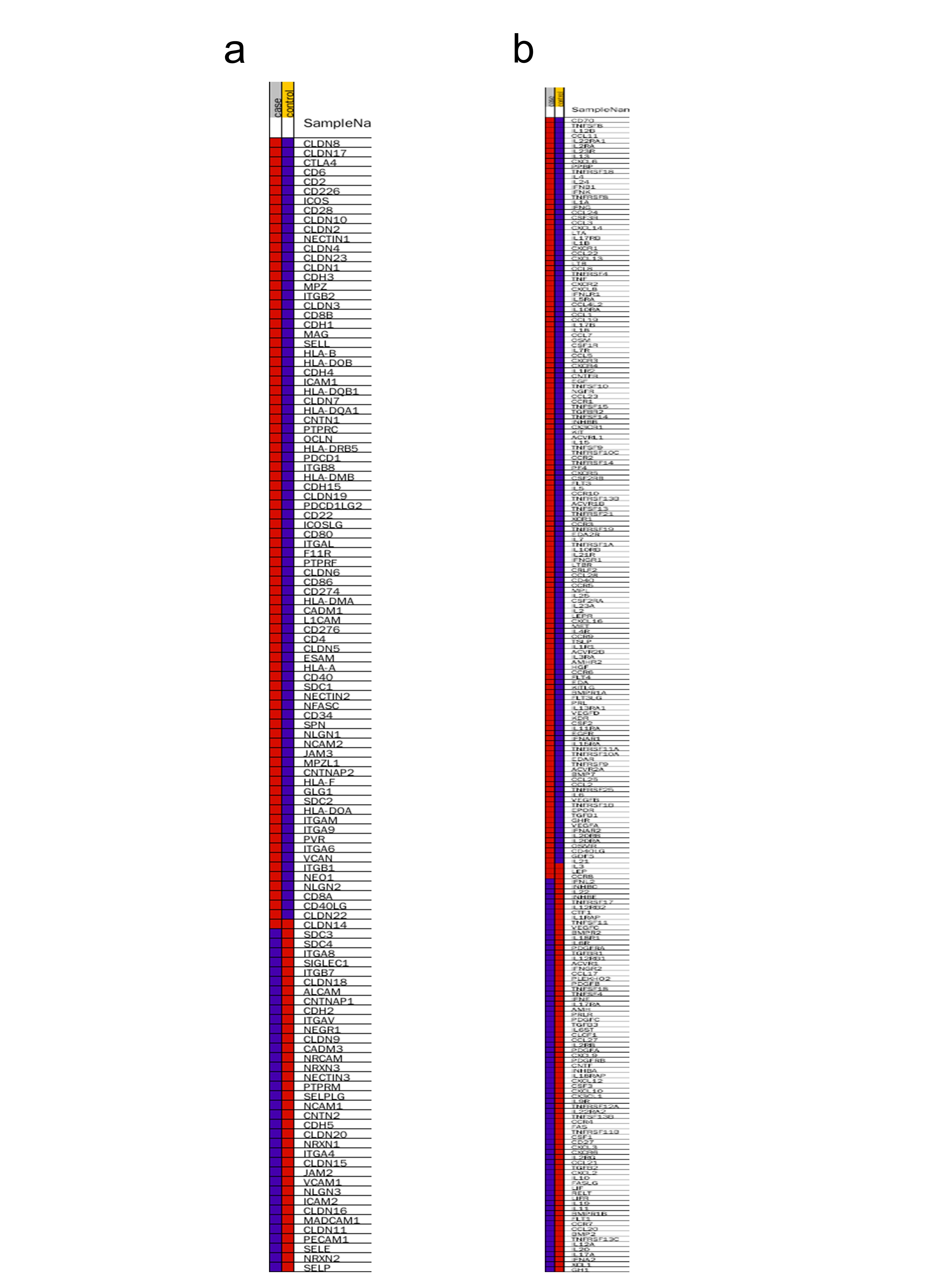
**

**Figure S4.** Genes enriched in KO and WT groups of GSEA. a. “Cell adhesion molecule” pathway enriched genes of tumor cells. b. “Cytokine-cytokine receptor interaction signal pathway” enriched genes of tumor cells.

**
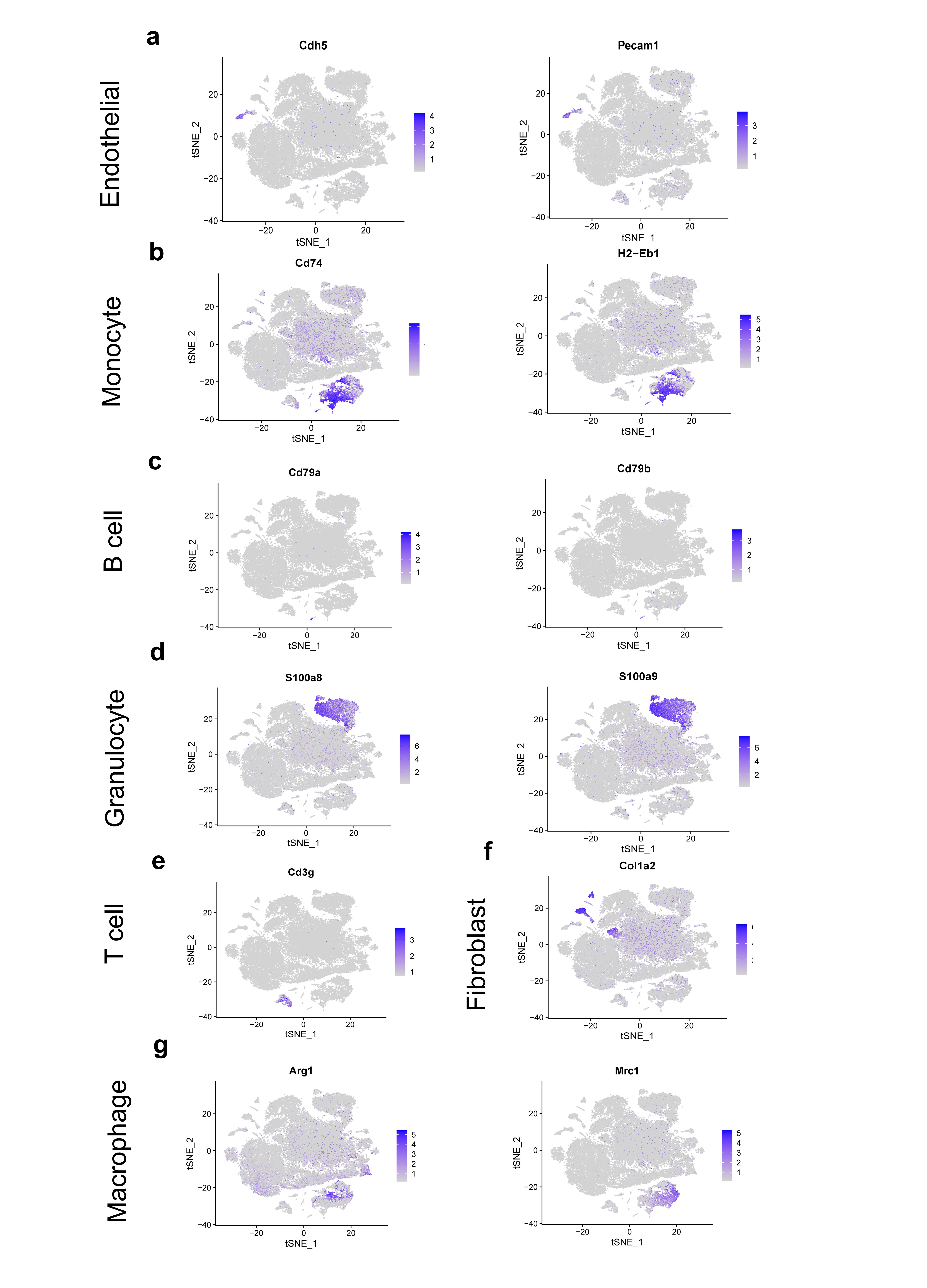
**

**Figure S5. Marker genes of six stromal cells.** a. Marker genes of endothelial were shown by t-SNE. b. Marker genes of monocyte were shown by t-SNE. c. Marker genes of B cell were shown by t-SNE. d. Marker genes of granulocyte were shown by t-SNE. e. Marker gene of T cell was shown by t-SNE. f. Marker gene of fibroblast was shown by t-SNE. g. Marker gene of macrophage were shown by t-SNE


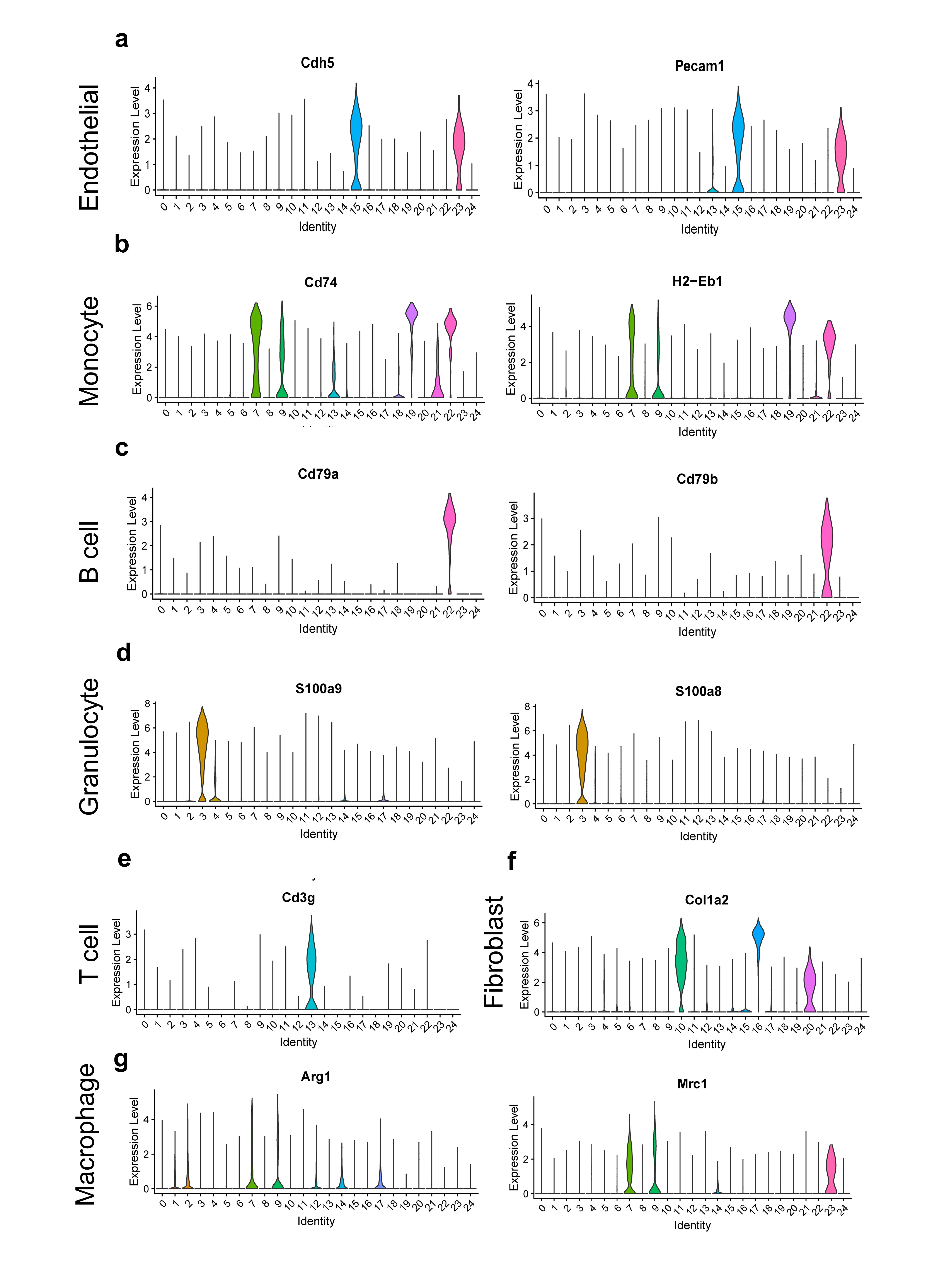


**Figure S6. Marker genes expression of six stromal cells.** a. Marker genes expression of endothelial in violin plots. b. Marker genes expression of monocyte in violin plots. c. Marker genes expression of B cell in violin plots. d. Marker genes expression of granulocyte in violin plots. e. Marker genes expression of T cell in violin plot. f. Marker genes expression of fibroblast was shown in violin plot. g. Marker genes expression of macrophage were shown in violin plots.


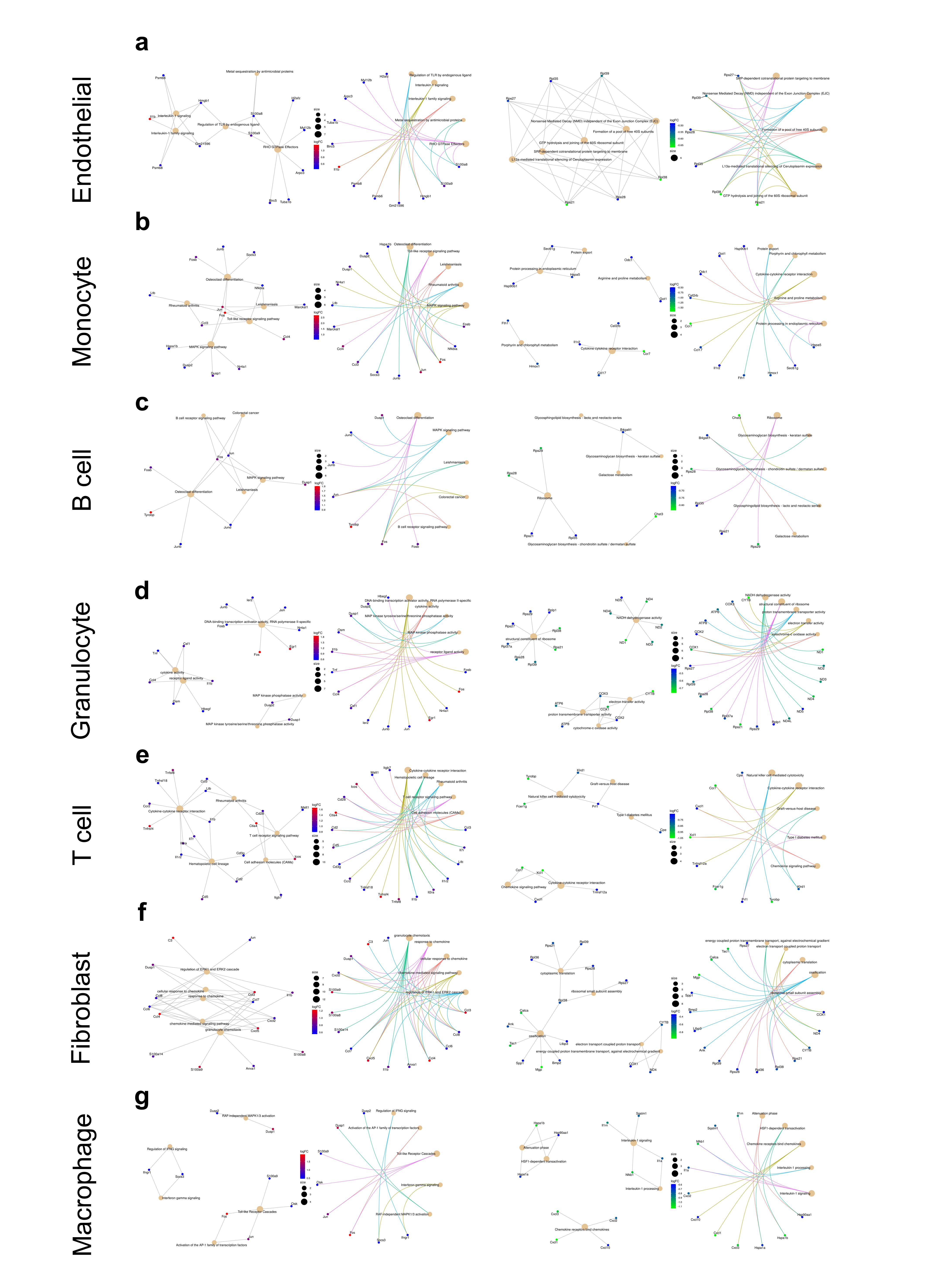


**Figure S7. Functional enrichment analysis of six stromal cells.** a-g. KEGG result shows the enrichment signal pathway based on positive top50 expressed gene in KO group and negative top50 expressed gene in KO group in endothelial, monocyte, B cell, granulocyte, T cell, fibroblast and macrophage.


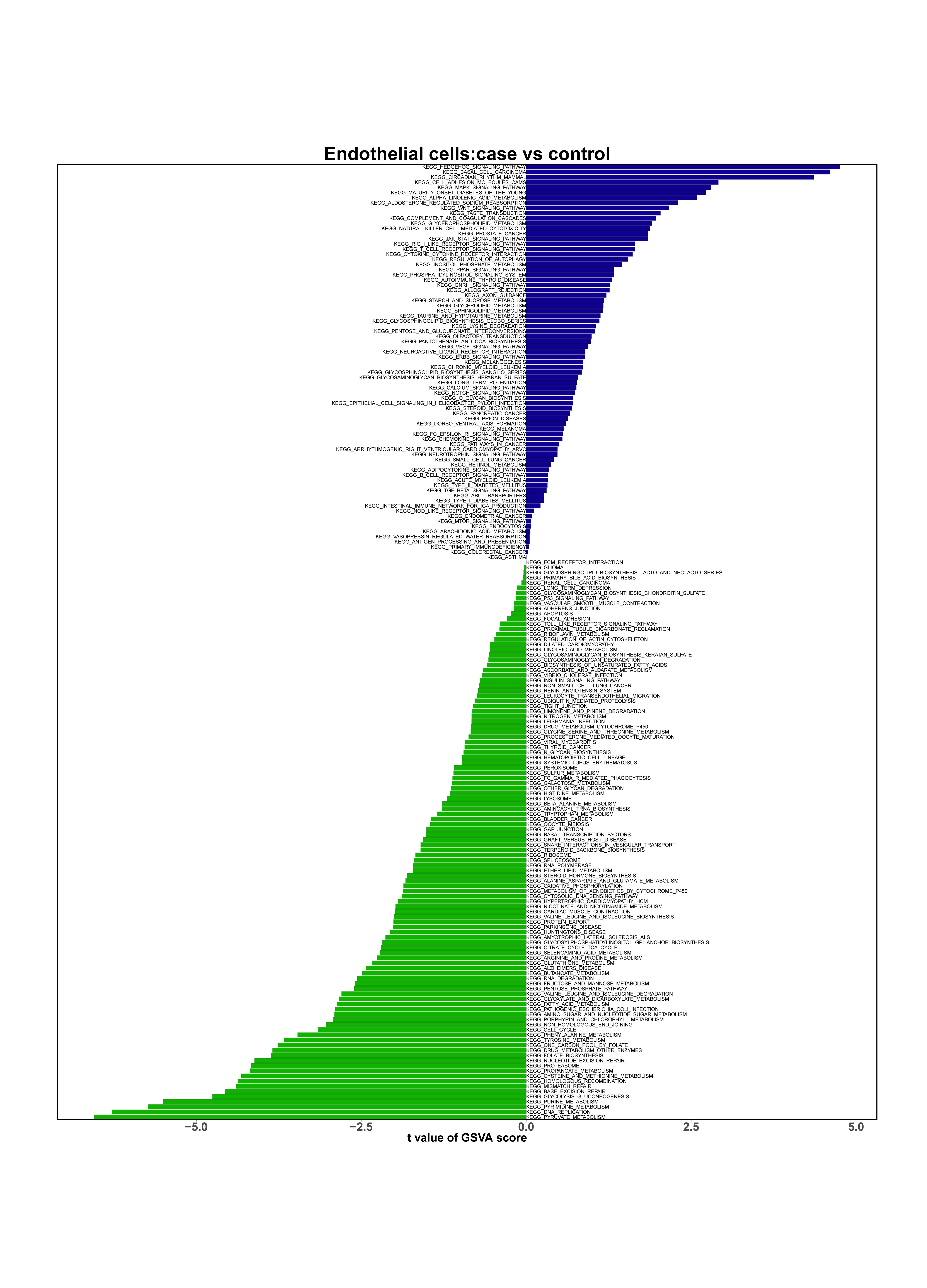


**Figure S8. GSVA analysis of endothelial.** GSVA analysis based on KEGG pathways between KO and WT group of endothelial. Blue represents KO group, green represents WT group.


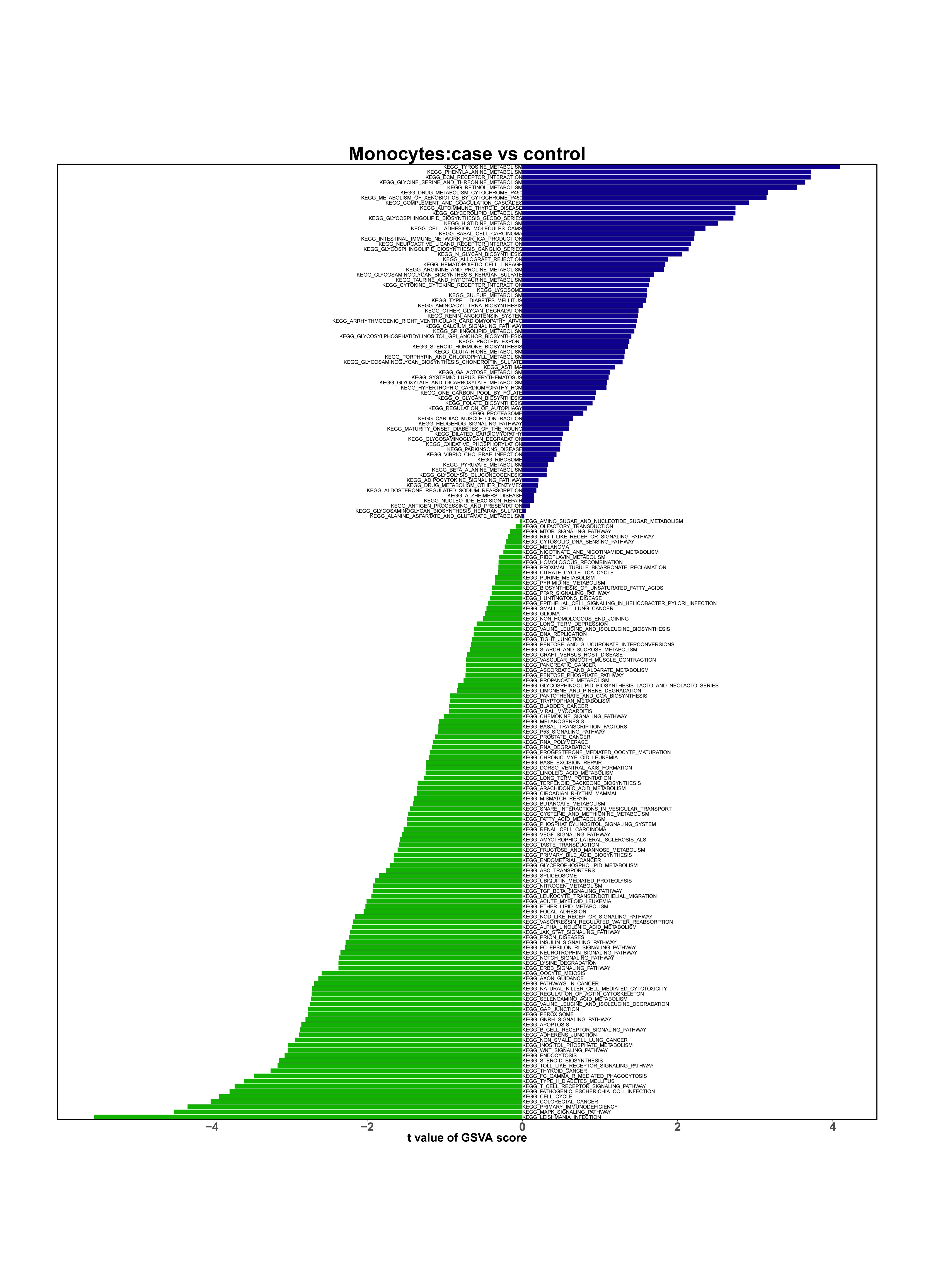


**Figure S9. GSVA analysis of monocyte.** GSVA analysis based on KEGG pathways between KO and WT group of monocyte. Blue represents KO group, green represents WT group.


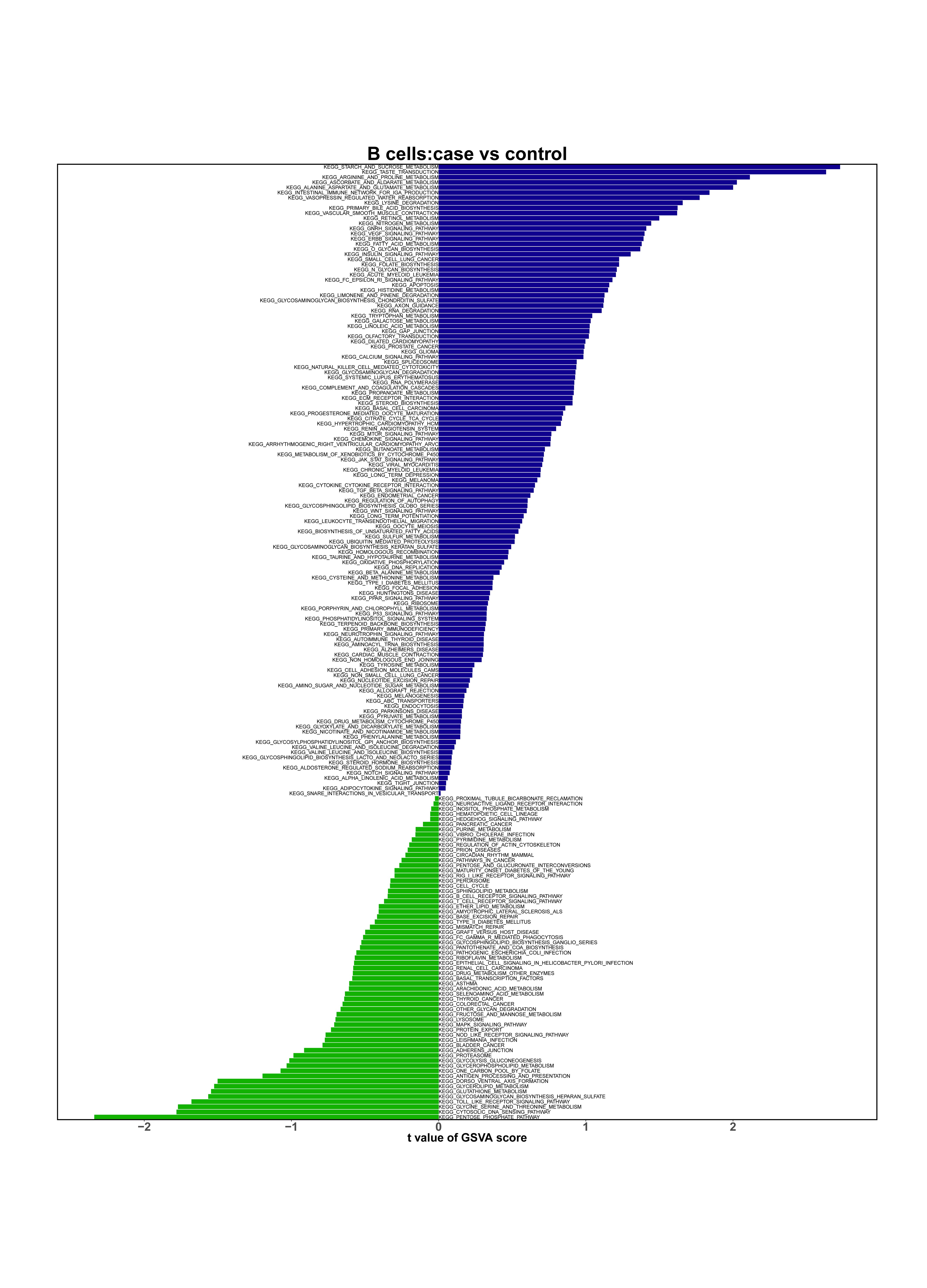


**Figure S10. GSVA analysis of B cell.** GSVA analysis based on KEGG pathways between KO and WT group of B cell. Blue represents KO group, green represents WT group.


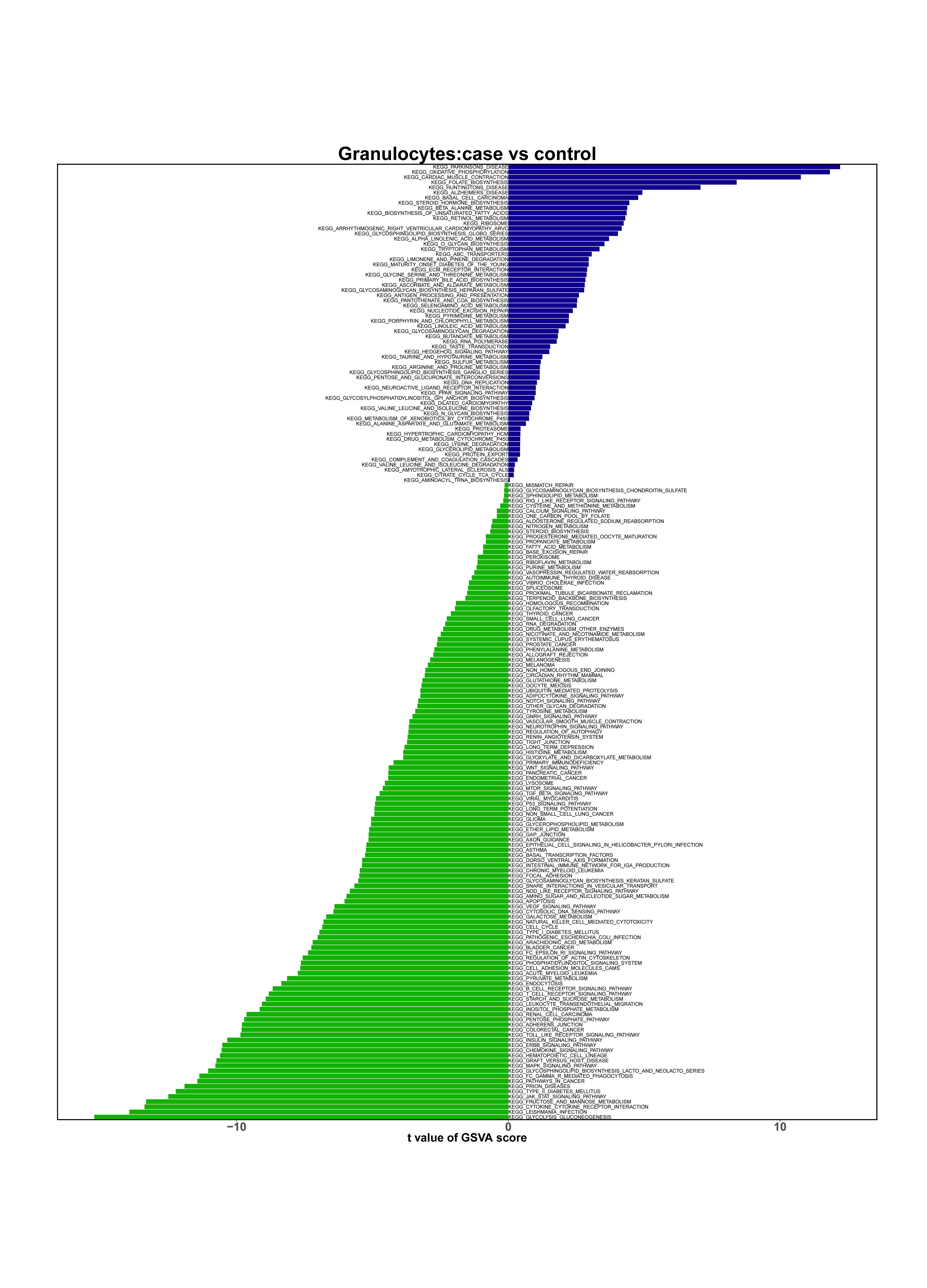


**Figure S11. GSVA analysis of granulocyte.** GSVA analysis based on KEGG pathways between KO and WT group of granulocyte. Blue represents KO group, green represents WT group.


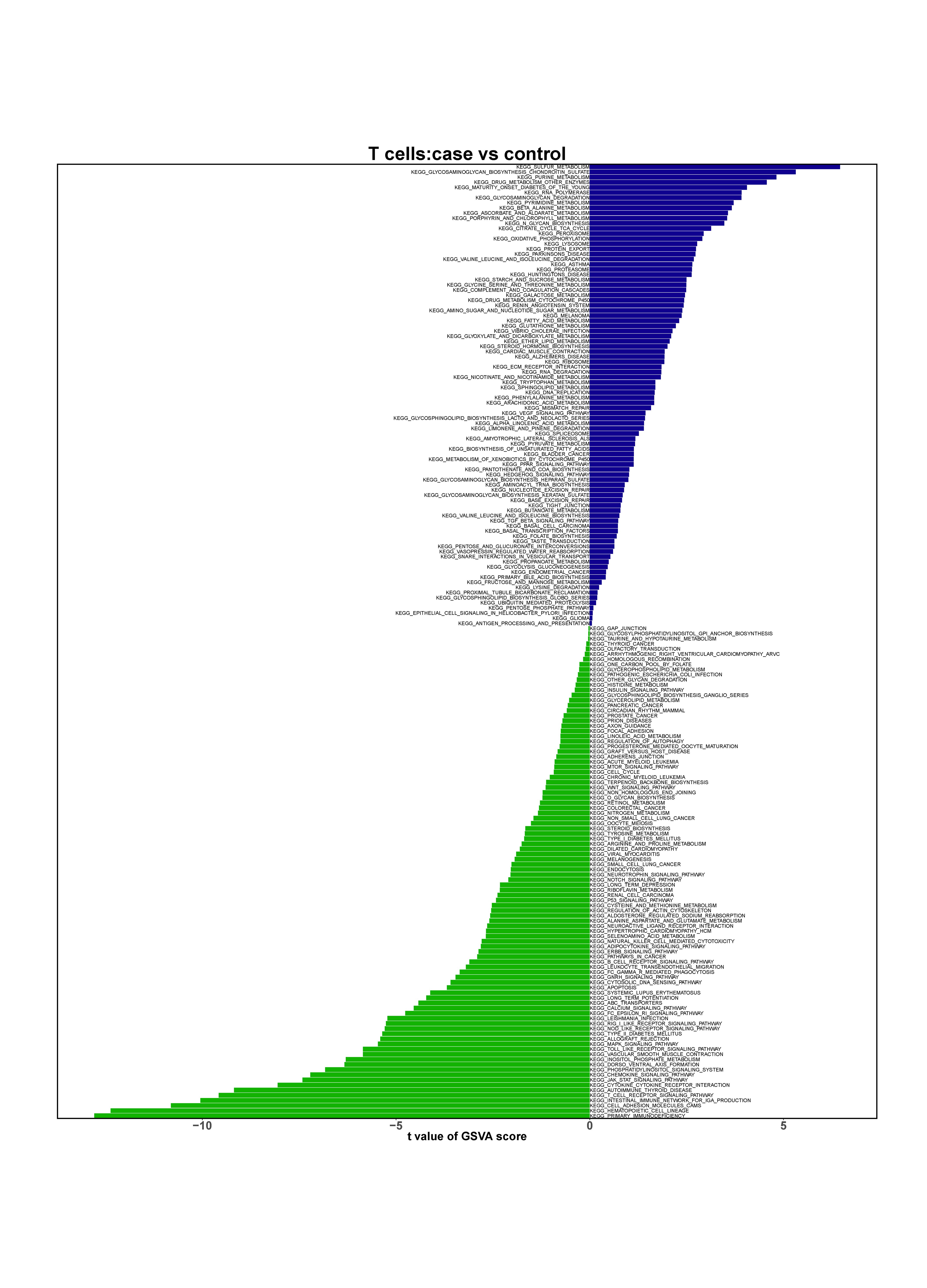


**Figure S12. GSVA analysis of T cell.** GSVA analysis based on KEGG pathways between KO and WT group of T cell. Blue represents KO group, green represents WT group.


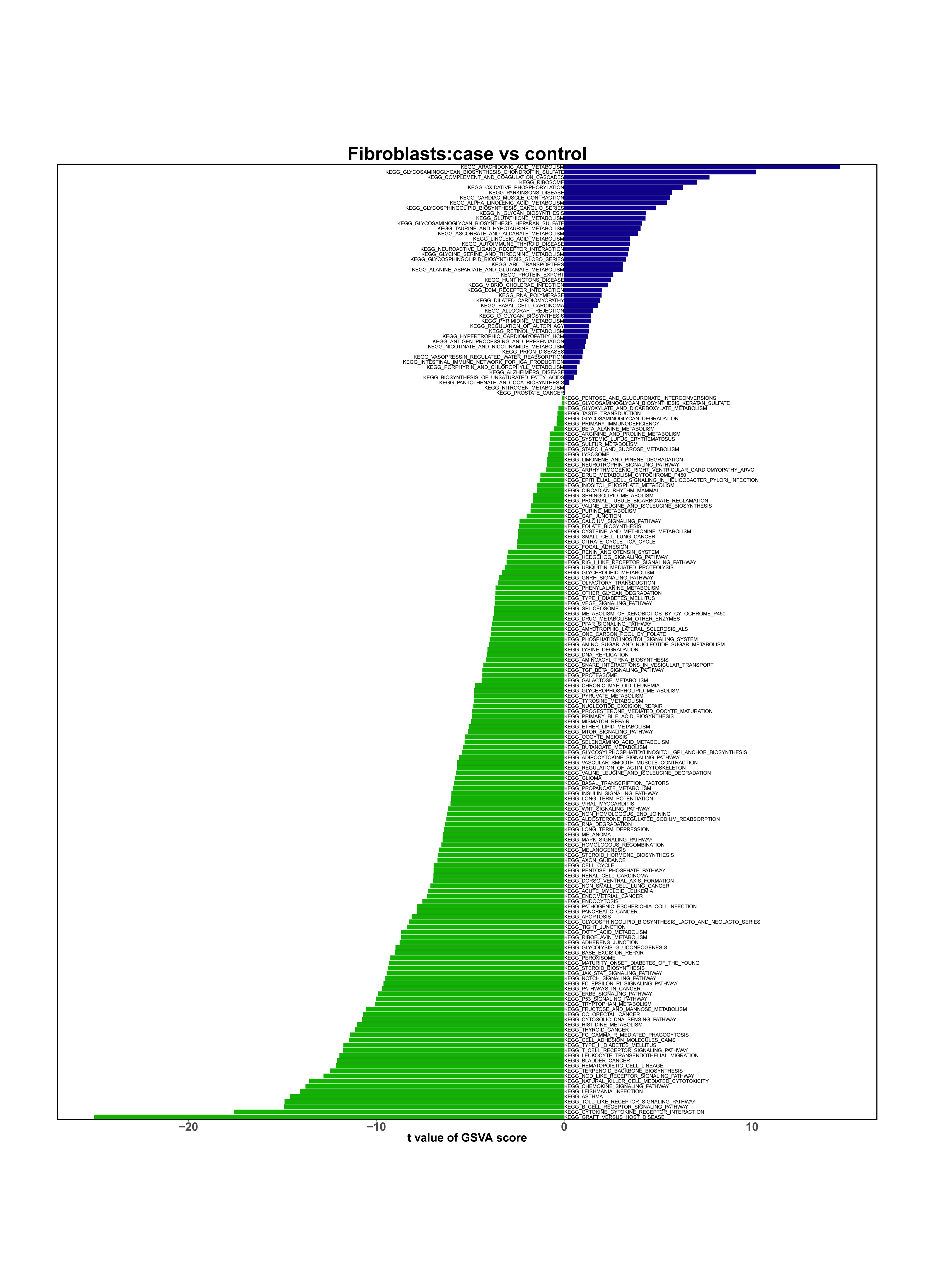


**Figure S13. GSVA analysis of fibroblast.** GSVA analysis based on KEGG pathways between KO and WT group of fibroblast. Blue represents KO group, green represents WT group.


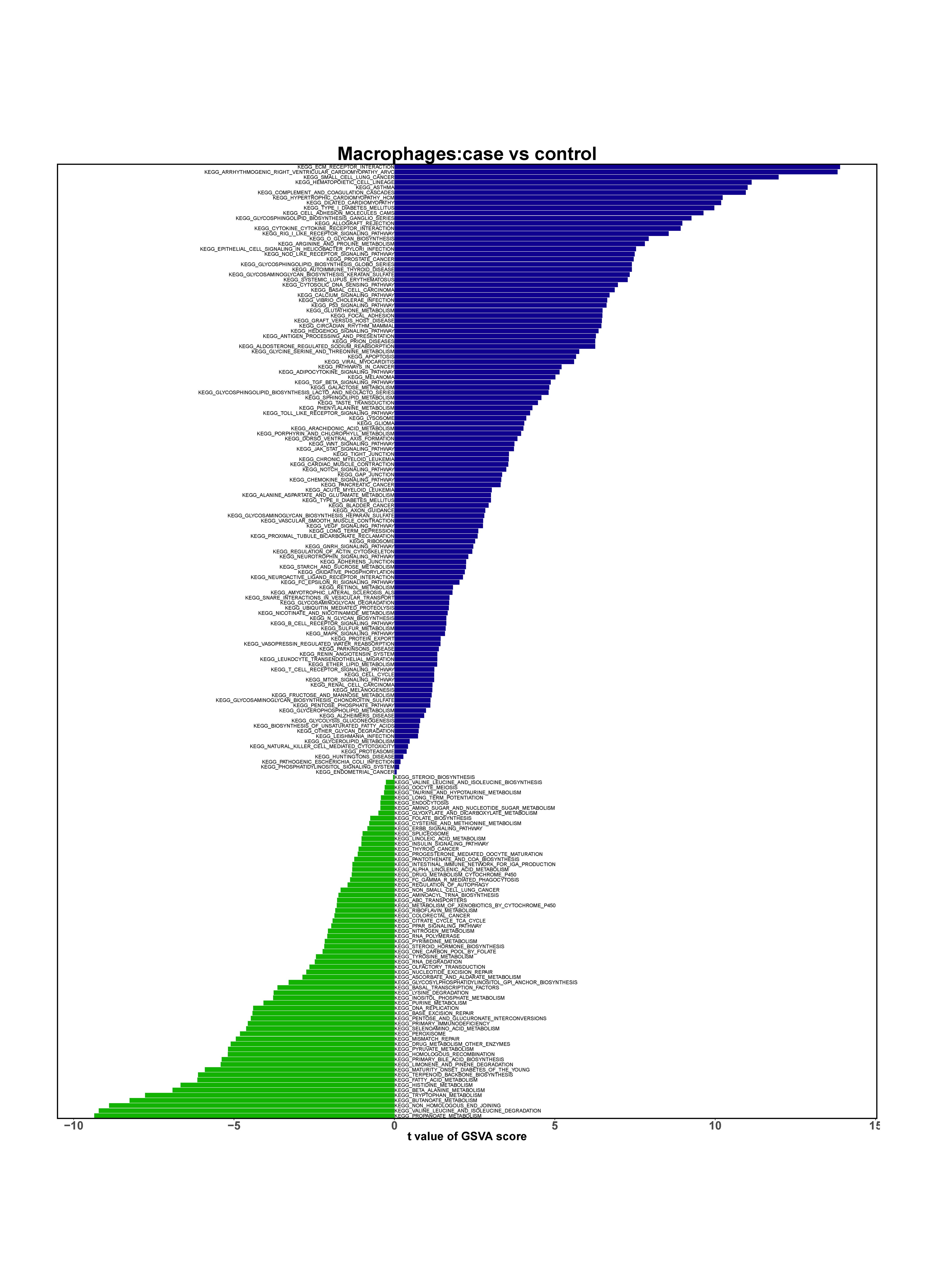


**Figure S14. GSVA analysis of macrophage.** GSVA analysis based on KEGG pathways between KO and WT group of macrophage. Blue represents KO group, green represents WT group.


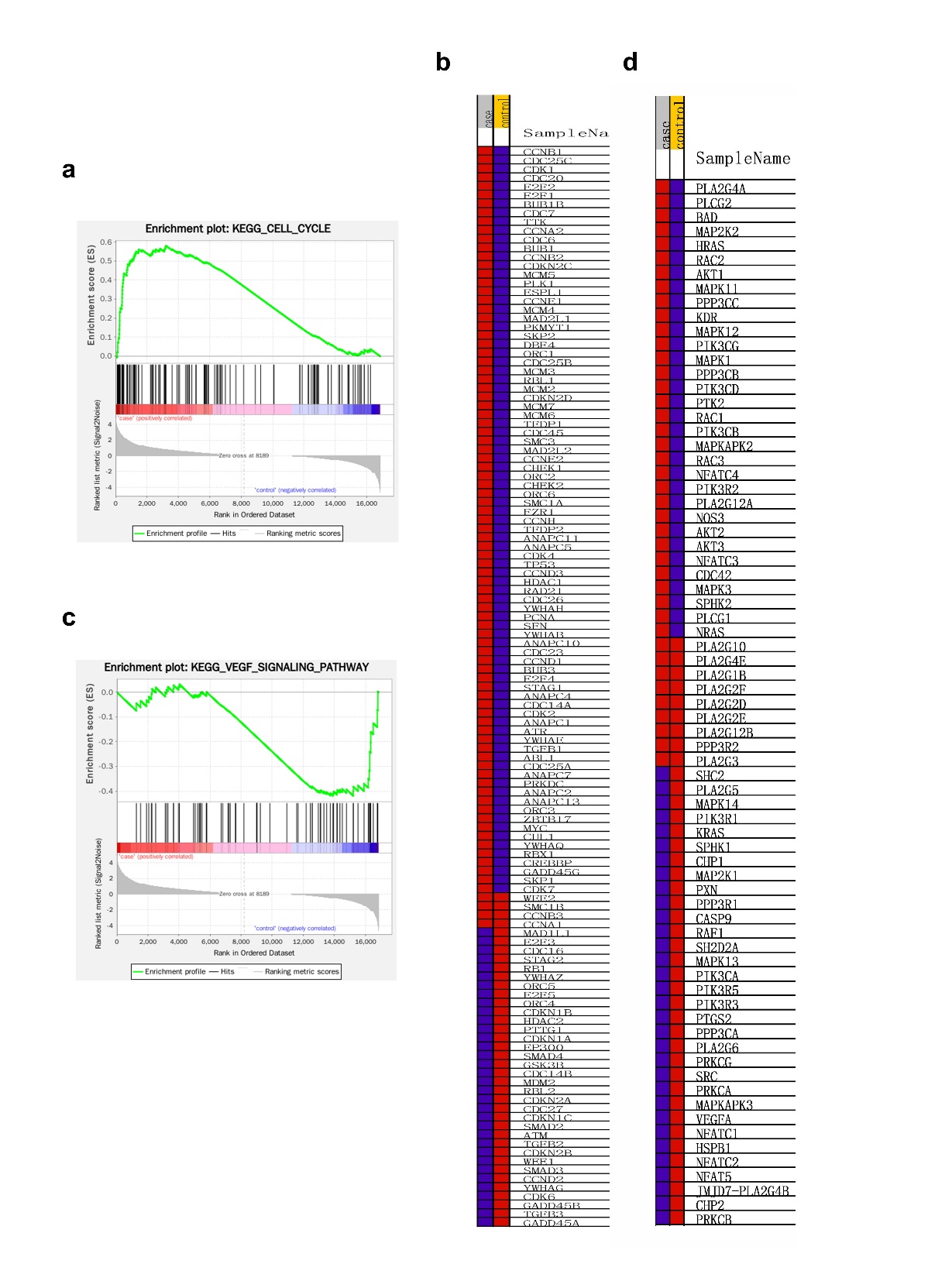


**Figure S15. GSEA analysis of endothelials.** a. The regulation trend of “Cell-cycle signaling pathway” between KO and WT group in endothelial. b. Enriched genes of “Cell-cycle signaling pathway” between KO and WT group. c. The regulation trend of “VEGF signal pathway” between KO and WT group. d. Enriched genes of “VEGF signal pathway” between KO and WT group.


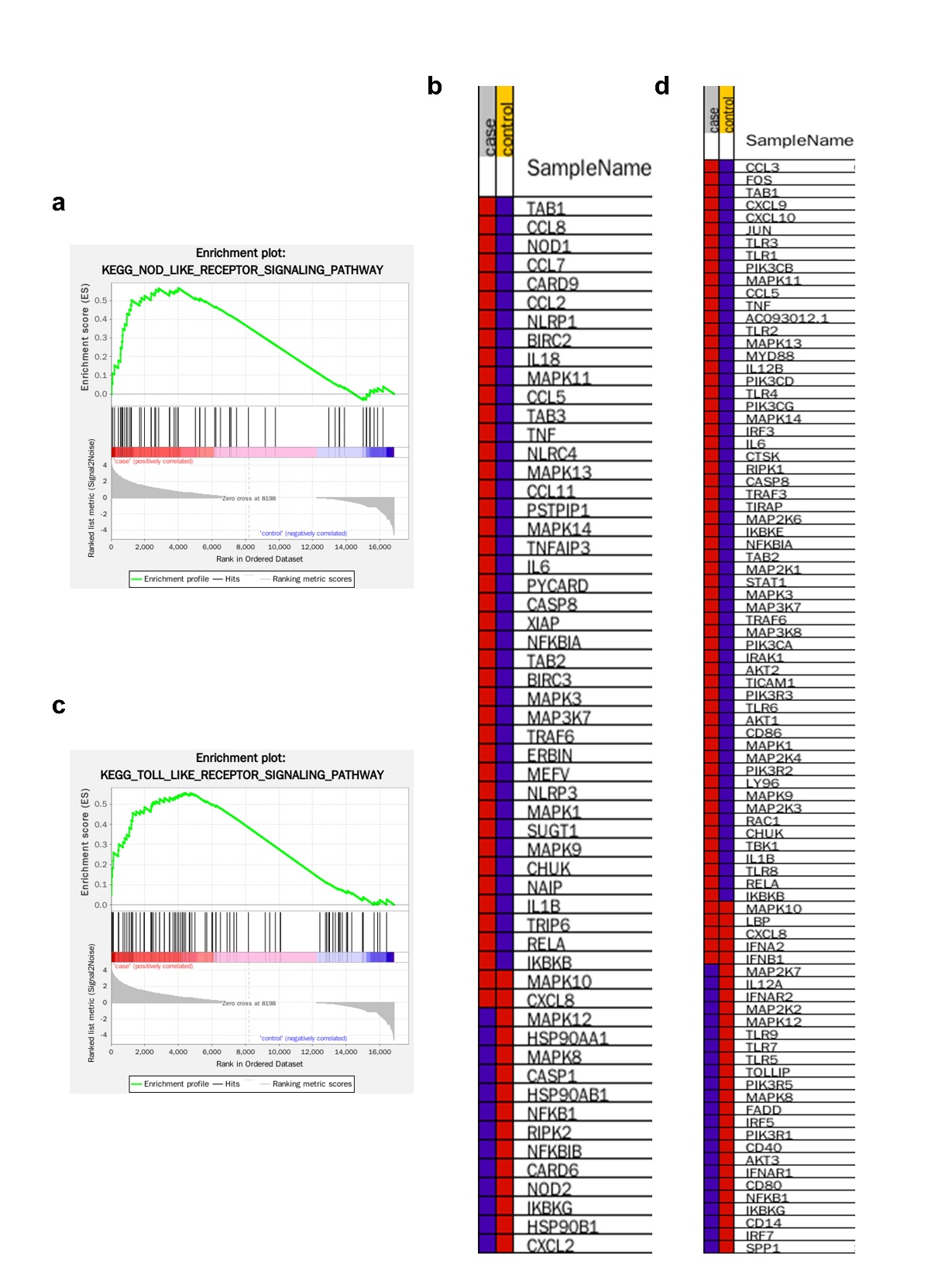


**Figure S16. GSEA analysis of monocytes.** a. The regulation trend of “Toll-like signaling pathway” between KO and WT group in monocyte. b. Enriched genes of “Toll-like signaling pathway” between KO and WT group. c. The regulation trend of “Nod-like signaling pathway”between KO and WT group. d. Enriched genes of “VEGF signal pathway” between KO and WT group.


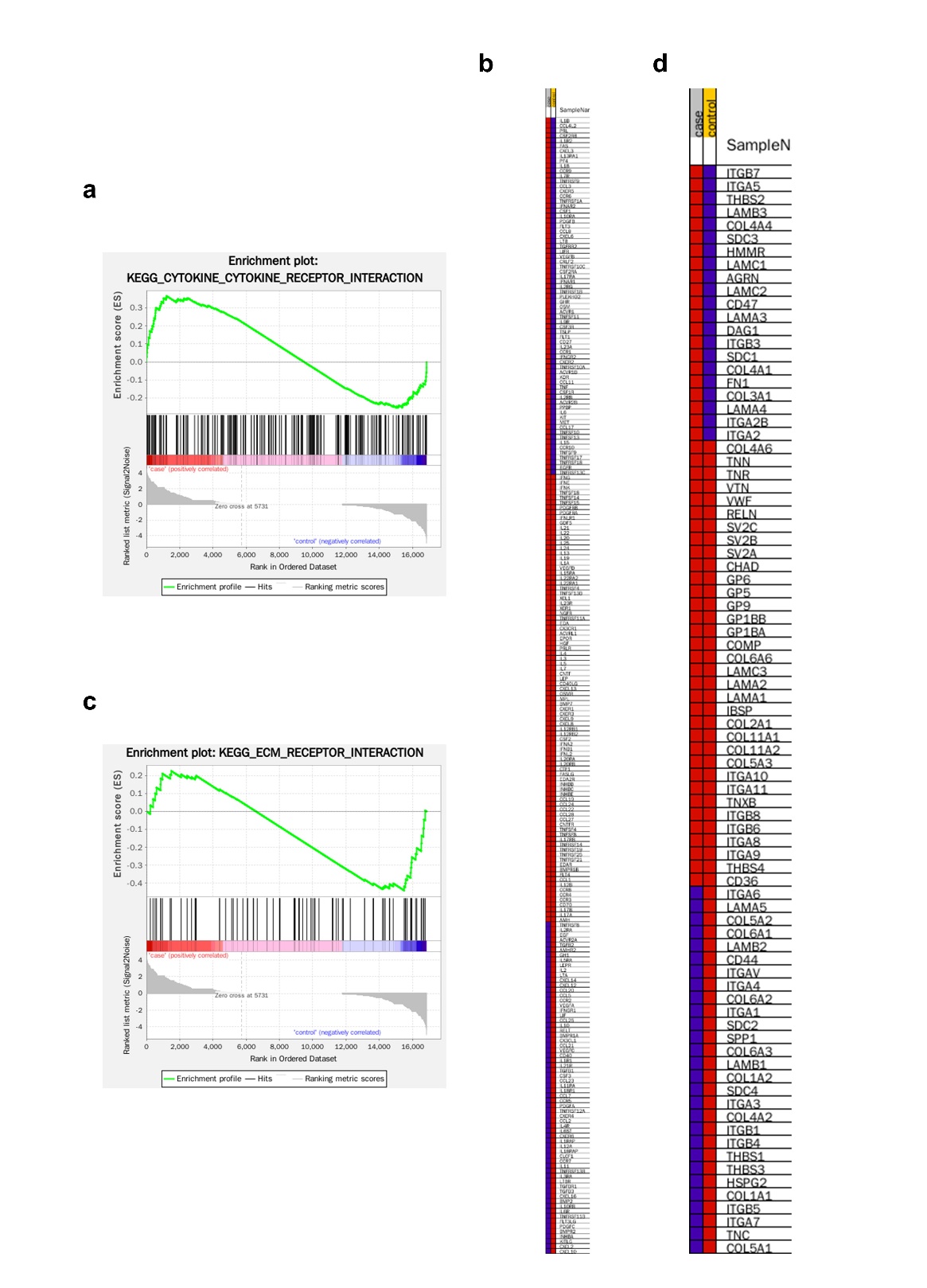


**Figure S17. GSEA analysis of B cells.** a. The regulation trend of “Cytokine-cytokine receptor interaction” between KO and WT group in B cells. b. Enriched genes of “Cytokine-cytokine receptor interaction” between KO and WT group. c. The regulation trend of “ECM receptor interaction signal pathway” between KO and WT group. d. Enriched genes of “ECM receptor interaction signal pathway” between KO and WT group.


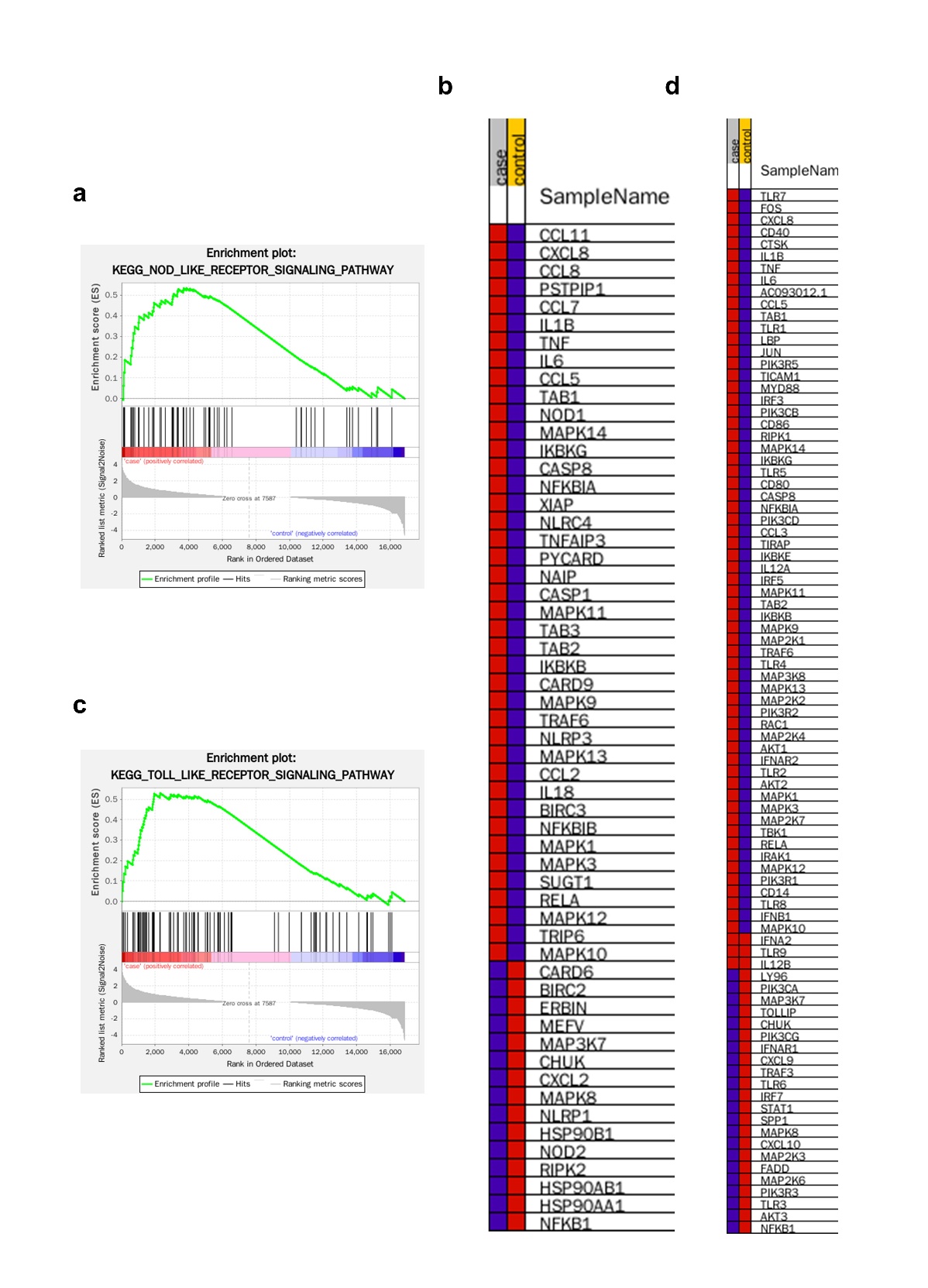


**Figure S18. GSEA analysis of granulocytes.** a. The regulation trend of “Nod-like receptor signaling pathway” between KO and WT group in granulocyte. b. Enriched genes of “Nod-like receptor signaling pathway” between KO and WT group. c. The regulation trend of “Toll-like receptor signaling pathway” between KO and WT group. d. Enriched genes of “Toll-like receptor signaling pathway” between KO and WT group.


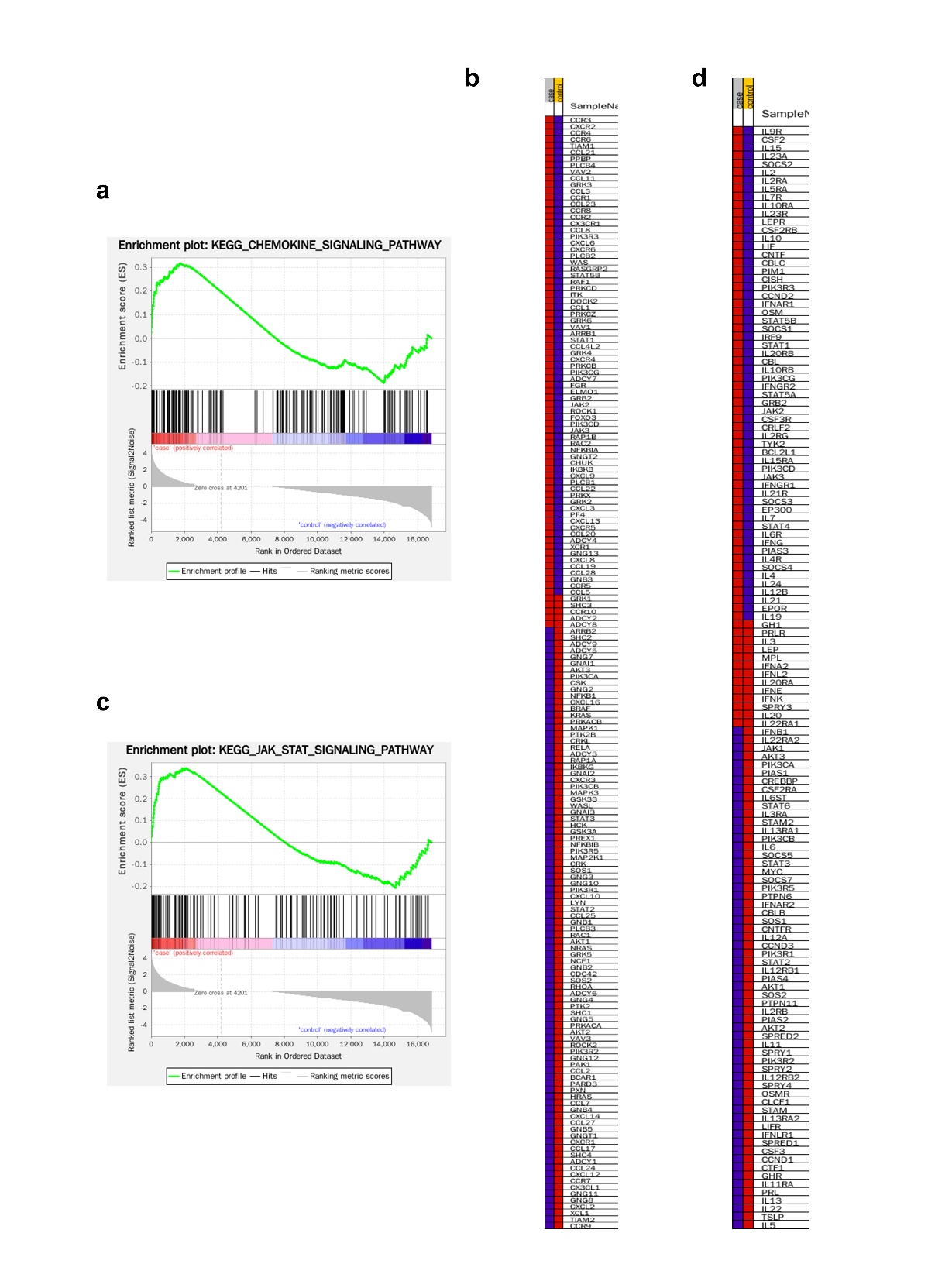


**Figure S19. GSEA analysis of T cells.** a. The regulation trend of “Chemokine signaling pathway” between KO and WT group in T cells. b. Enriched genes of “Chemokine signaling pathway” between KO and WT group. c. The regulation trend of “JAK-STAT signaling pathway” between KO and WT group. d. Enriched genes of “JAK-STAT signaling pathway” between KO and WT group.


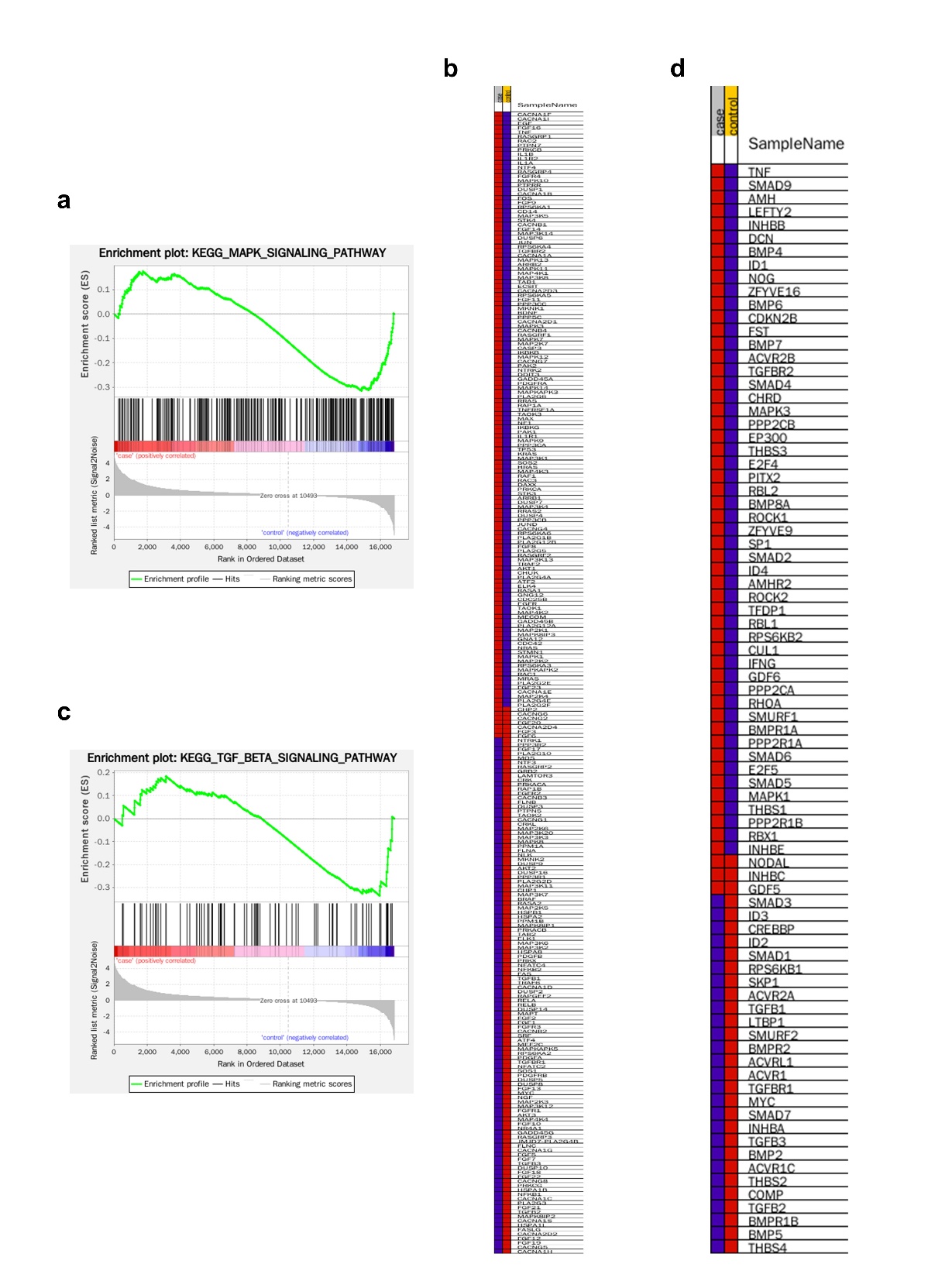


**Figure S20. GSEA analysis of fibroblasts.** a. The regulation trend of “TGF-β signaling pathway” between KO and WT group in fibroblast. b. Enriched genes of “TGF-β signaling pathway” between KO and WT group. c. The regulation trend of “MAPK signaling pathway” between KO and WT group. d. Enriched genes of “MAPK signaling pathway” between KO and WT group.


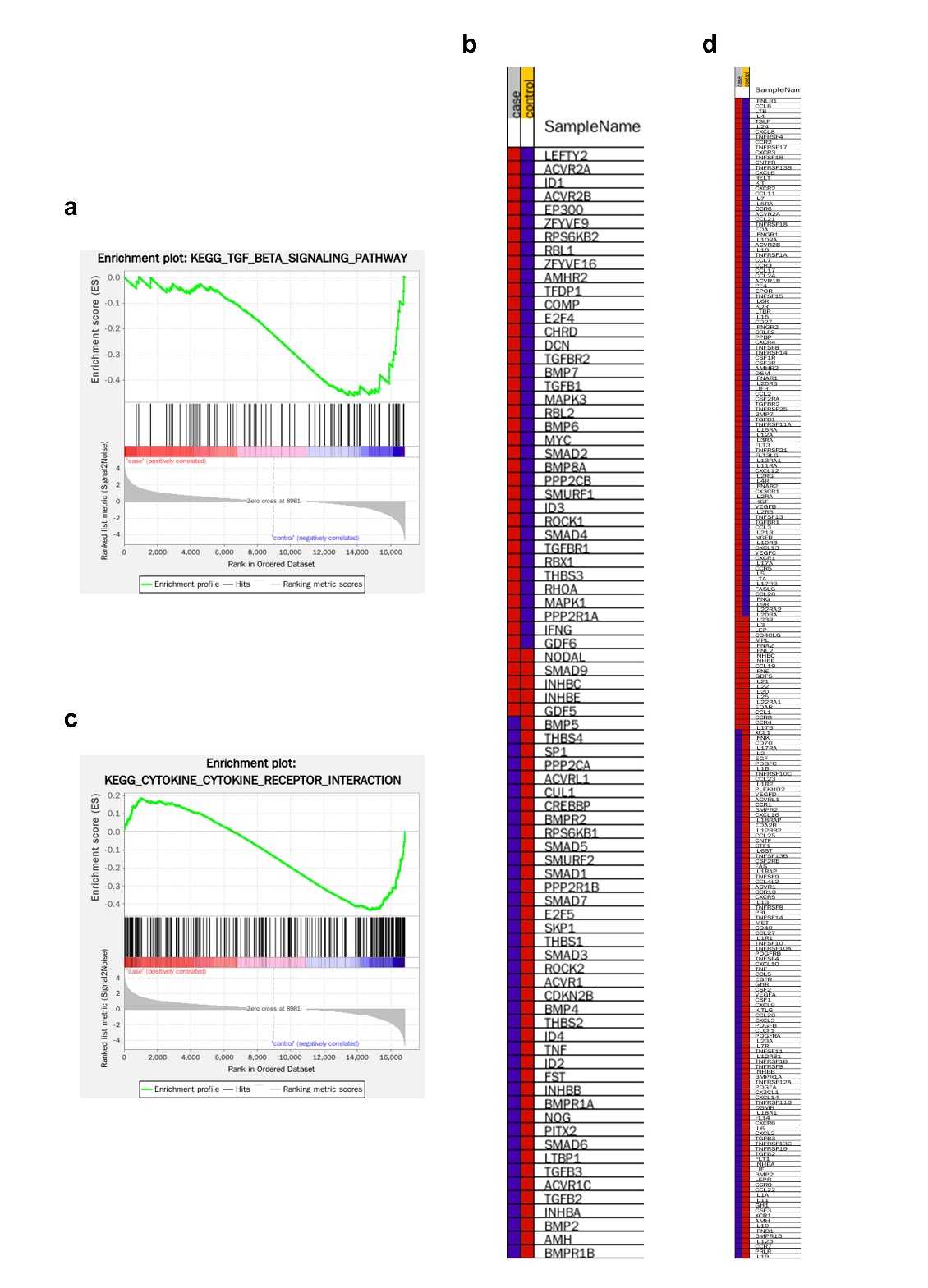


**Figure S21. GSEA analysis of macrophages.** a. The regulation trend of “TGF-β signaling pathway” between KO and WT group in macrophage. b. Enriched genes of “TGF-β signaling pathway” between KO and WT group. c. The regulation trend of “Chemokine receptor signaling pathway” between KO and WT group. d. Enriched genes of “Chemokine receptor signaling pathway” between KO and WT group.


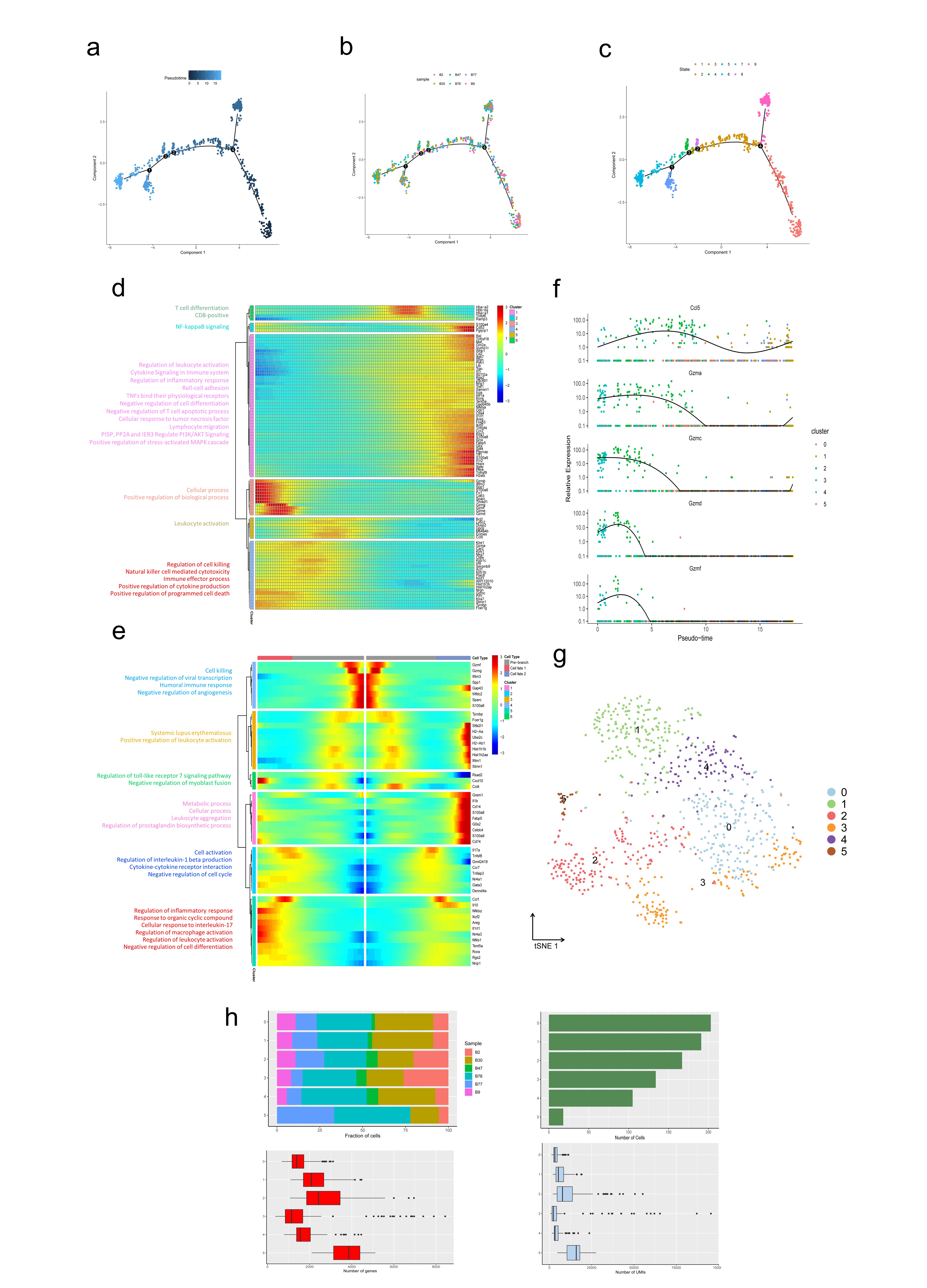


**Figure S22. Trajectory analysis and re-cluster of T cells.** a-c. Trajectory analysis diagrams of pseudotime in different sampls and different states were shown. d. The top100 differential gene function enrichment heatmap. e. The differential expression genes of different branches and GO BP pathways of different clusters were shown in heatmap. f. The Dynamic changes of top5 differential expression genes determined cell fate between different clusters were shown. g. Subcluster of T cells were shown by t-SNE. h. The fraction of cells in different clusters among six samples, number of cells in different clusters and number of genes in different clusters were shown.


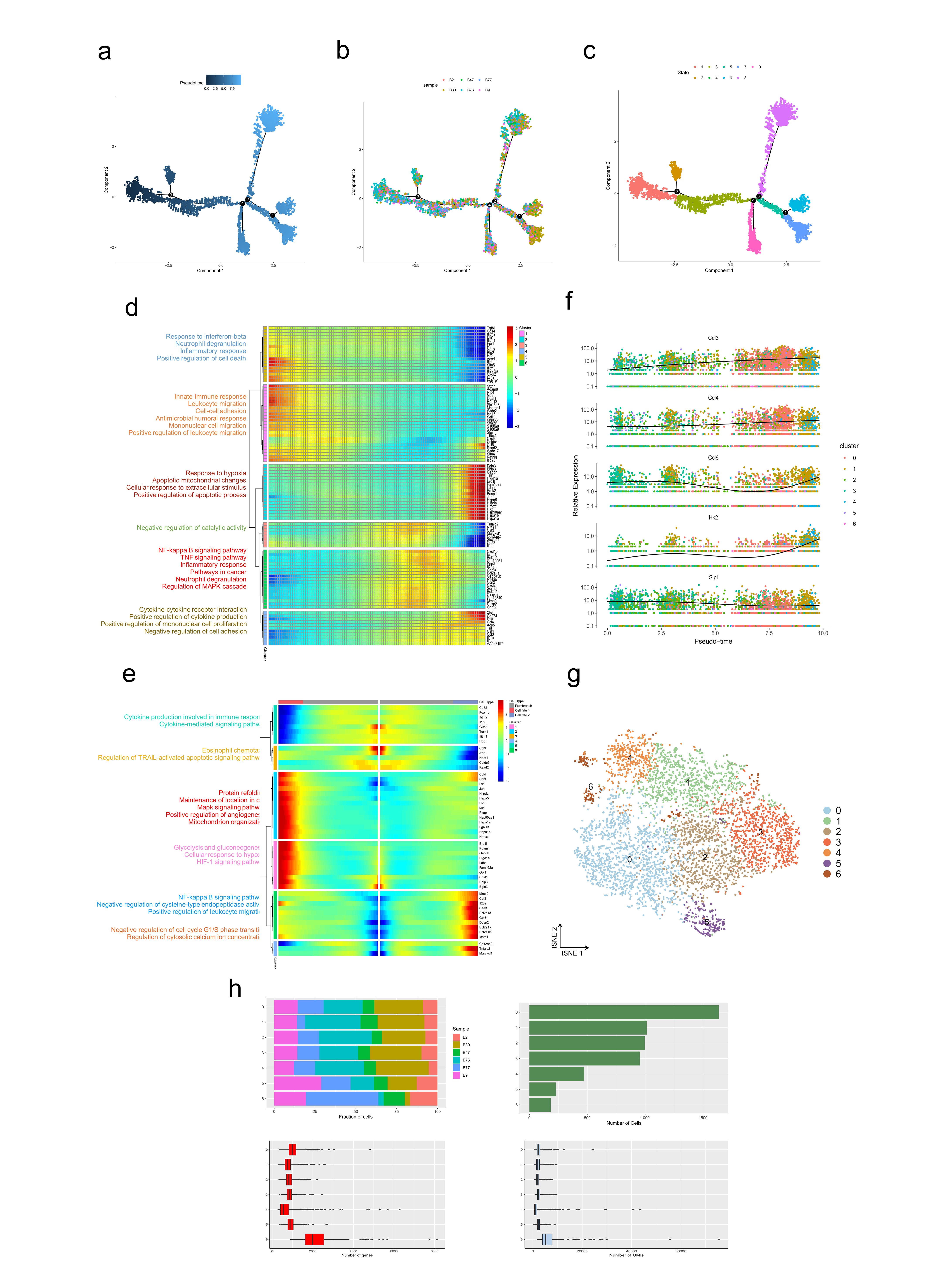


**Figure S23. Trajectory analysis and re-cluster of granulocytes.** a-c. Trajectory analysis diagrams of pseudotime in different sampls and different states were shown. d. The top100 differential gene function enrichment heatmap. e. The differential expression genes of different branches and GO BP pathways of different clusters were shown in heatmap. f. The Dynamic changes of top5 differential expression genes determined cell fate between different clusters were shown. g. Subcluster of granulocytes were shown by t-SNE. h. The fraction of cells in different clusters among six samples, number of cells in different clusters and number of genes in different clusters were shown.

**
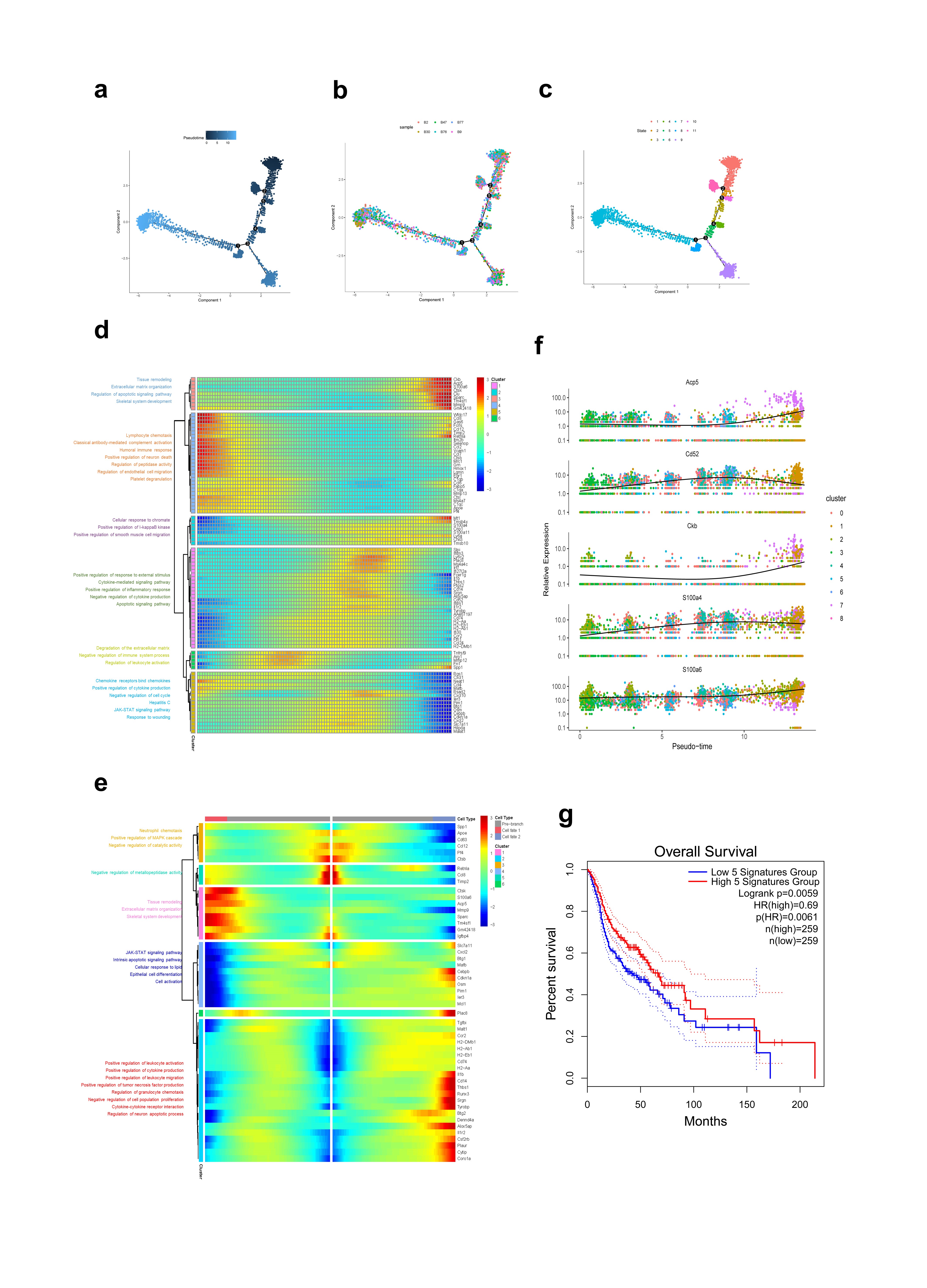
**

**Figure S24. Trajectory analysis of macrophages.** a-c. Trajectory analysis diagrams of pseudotime in different sampls and different states were shown. d. The top100 differential gene function enrichment heatmap. e. The differential expression genes of different branches and GO BP pathways of different clusters were shown in heatmap. f. The Dynamic changes of top5 differential expression genes determined cell fate between different clusters were shown. g. Survival analysis of OSCC gene features using TCGA-PAAD based on Fig S24f on the Gepia2 website (http:// gepia2. cancer- pku. cn/# index).

Table S1 Primer sequences used in RT-PCR

| Primer | Sequence（5’-3’） |
| --- | --- |
| CD206 Forward | 5'-CCATGGACAATGCGCGAGCG-3' |
| CD206 Reverse | 5'-CACCTGTGGCCCAAGACACGT-3' |
| TGF-β Forward | 5'- CAAGTGGACATCAACGGGTTC -3' |
| TGF-β Reverse | 5'- GCCATGAGAAGCAGGAAAGG -3' |
| ARG-1 Forward | 5'- TTGGCTTGAGAGACGTGGAC -3' |
| ARG-1 Reverse | 5'- GTGCCAGTAGCTGGTGTGAA -3' |
| IL-10 Forward | 5'-CCAAGACCCAGACATCAAGG-3' |
| IL-10 Reverse | 5'-AAGGCATTCTTCACCTGCTC-3' |
| GAPDH Forward | 5'-ATCCCATCACCATCTTCC-3' |
| GAPDH Reverse | 5'-GAGTCCTTCCACGATACCA-3' |
| Dusp1 Forward | 5'-TCAAAGGAGGATACGAAG-3' |
| Dusp1 Reverse | 5'-ACTGCCCAGGTACAGAAA-3' |

Table S2 Proportion of different cell types in KO group and WT group

| Cell type | KO (means±SD) | WT (means±SD) | *P-value* |
| --- | --- | --- | --- |
| Tumor cells | 0.39±0.07 | 0.49±0.09 | 0.27 |
| Fibroblasts | 0.35±0.05 | 0.37±0.09 | 0.84 |
| Granulocytes | 0.13±0.04 | 0.06±0.01 | 0.06 |
| Macrophages | 0.09±0.03 | 0.06±0.002 | 0.27 |
| T cells | 0.02±0.007 | 0.007±0.003 | 0.06 |
| Endothelial cells | 0.013±0.004 | 0.010±0.005 | 0.51 |
| Monocytes | 0.006±0.001 | 0.003±0.001 | 0.046 |
| B cells | 0.0001±0.0001 | 0.0019±0.0006 | 0.61 |
